# The resistomes of six carbapenem-resistant pathogens – a critical genotype–phenotype analysis

**DOI:** 10.1099/mgen.0.000233

**Published:** 2018-11-21

**Authors:** Anna Johnning, Nahid Karami, Erika Tång Hallbäck, Vilhelm Müller, Lena Nyberg, Mariana Buongermino Pereira, Callum Stewart, Tobias Ambjörnsson, Fredrik Westerlund, Ingegerd Adlerberth, Erik Kristiansson

**Affiliations:** ^1^​ Department of Mathematical Sciences, Chalmers University of Technology, Gothenburg, Sweden; ^2^​ Centre for Antibiotic Resistance Research, CARe, University of Gothenburg, Gothenburg, Sweden; ^3^​ Department of Infectious Diseases, Institute of Biomedicine, Sahlgrenska Academy, University of Gothenburg, Gothenburg, Sweden; ^4^​ Department of Biology and Biological Engineering, Chalmers University of Technology, Gothenburg, Sweden; ^5^​ Department of Astronomy and Theoretical Physics, Lund University, Lund, Sweden

**Keywords:** whole-genome sequencing, carbapenem resistance, genotype–phenotype association, human pathogens

## Abstract

Carbapenem resistance is a rapidly growing threat to our ability to treat refractory bacterial infections. To understand how carbapenem resistance is mobilized and spread between pathogens, it is important to study the genetic context of the underlying resistance mechanisms. In this study, the resistomes of six clinical carbapenem-resistant isolates of five different species – *Acinetobacter baumannii*, *Escherichia coli*, two *Klebsiella pneumoniae*, *Proteus mirabilis* and *Pseudomonas aeruginosa* – were characterized using whole genome sequencing. All *Enterobacteriaceae* isolates and the *A. baumannii* isolate had acquired a large number of antimicrobial resistance genes (7–18 different genes per isolate), including the following encoding carbapenemases: *bla*_KPC-2_, *bla*_OXA-48_, *bla*_OXA-72_, *bla*_NDM-1_, *bla*_NDM-7_ and *bla*_VIM-1_. In addition, a novel version of *bla*_SHV_ was discovered. Four new resistance plasmids were identified and their fully assembled sequences were verified using optical DNA mapping. Most of the resistance genes were co-localized on these and other plasmids, suggesting a risk for co-selection. In contrast, five out of six carbapenemase genes were present on plasmids with no or few other resistance genes. The expected level of resistance – based on acquired resistance determinants – was concordant with measured levels in most cases. There were, however, several important discrepancies for four of the six isolates concerning multiple classes of antibiotics. In conclusion, our results further elucidate the diversity of carbapenemases, their mechanisms of horizontal transfer and possible patterns of co-selection. The study also emphasizes the difficulty of using whole genome sequencing for antimicrobial susceptibility testing of pathogens with complex genotypes.

## Data Summary

The assembled genomes have been deposited in GenBank under the following accession numbers: *Pseudomonas aeruginosa* CCUG 70744, CP023255.1; *E. coli* CCUG 70745.1, CP023258.1–CP023264.1; *K. pneumoniae* CCUG 70742, CP023249.1–CP0232453.1; *Proteus mirabilis* CCUG 70746.1, CP023273.1–CP023274.1; *A. baumannii* CCUG 70743, NTCX00000000 (the version described in this paper is NTCX00000000.1); and *K. pneumoniae* CCUG 70747, CP023441.1–CP023444.1. All raw sequencing data have been deposited in the Sequence Read Archive (SRA) and connected to BioProject PRJNA401330. Raw kymographs of individual plasmid molecules from the optical DNA mapping have been deposited with figshare (doi: 10.6084/m9.figshare.6455021.v1)

**Impact Statement**

The rapidly growing incidence of bacterial infections resisting antibiotic treatment is one of the largest threats to human health. This is particularly true for resistance against carbapenems – antibiotics often used as the drug of last resort for life-threatening bacterial infections. Studying the underlying genetic mechanisms of carbapenem resistance, and their means of transfer between bacteria, is therefore important for the implementation of management strategies and surveillance. Our results – based on genome analysis of six extensively drug-resistant pathogens – demonstrate the large diversity of carbapenemases encountered in clinical isolates. The observed co-localization of resistance genes on plasmids further elucidates how resistance is horizontally transferred between bacteria and the possible risks for co-selection. The combination of short- and long-read sequencing with optical DNA maps provided a novel way to experimentally validate assembled plasmid sequences and to detect structural variations overlooked by existing methodologies. Furthermore, our in-depth comparison between resistance genotypes and phenotypes showed important discrepancies, which demonstrate that there is still a lack of knowledge regarding resistance mechanisms. This pinpoints many of the difficulties in introducing whole-genome sequencing-based methods for antibiotic susceptibility testing of bacteria with more complex resistance profiles and the need for further studies of resistance mechanisms.

## Introduction

Carbapenems are broad-spectrum antibiotics and constitute one of our main weapons against multi-resistant bacteria. However, recent years have seen a rapid increase in carbapenem resistance globally [1]. Consequently, the World Health Organization has ranked carbapenem-resistant *Acinetobacter baumannii*, *Pseudomonas aeruginosa* and *Enterobacteriaceae* as critical threats to global health, and has requested increased research efforts regarding these pathogens [2]. Furthermore, carbapenem-resistant *Enterobacteriaceae* are regarded as one of the three most urgent drug resistance threats to the United States by the Centers for Disease Control and Prevention [3]. The increasing threat of carbapenem resistance is further underscored by statistics from the resistance surveillance network in Europe (EARS-net), which shows growing incidences of carbapenem-resistant infections by pathogens such as *Klebsiella pneumoniae*, *Acinetobacter* species and *Pseudomonas aeruginosa* [4].

The genetic mechanisms behind carbapenem resistance include efflux pumps, altered function or expression of porins and penicillin-binding proteins (PBPs), as well as the acquisition of enzymes capable of hydrolysing the drugs – so-called carbapenemases [5]. Many carbapenemases – especially those of class B – are capable of hydrolysing almost all known β-lactams while others have a narrower spectrum [6]. Yet clinical isolates with unknown mechanisms of carbapenem resistance are still being discovered [7, 8]. A key contributing factor to the rapid global spread of carbapenem resistance is transmissible plasmids that carry carbapenemase genes [9, 10]. Many of these resistance plasmids have a broad host range and are able to replicate and spread between distant taxonomic orders. Thus, to continue the characterization of carbapenem resistance factors and to assess the risk for horizontal transfer and dissemination of carbapenemase genes, it is important to study the genomes of pathogens carrying these genes. Furthermore, the recent cost reduction of whole-genome sequencing (WGS) has made it a proposed complement to traditional antimicrobial susceptibility testing [11, 12]. For WGS to be established as a generally viable option to the culture-based characterization of clinical isolates, the association between the resistance genotype and phenotype needs to be further studied. This is especially true for many carbapenem-resistant pathogens which often exhibit complex phenotypic profiles with resistance to multiple classes of antibiotics.

Carbapenemase genes are frequently encoded on plasmids or other mobile genetic elements that could potentially be carrying additional resistance genes against other classes of antibiotics. Analysis of the genetically linked co-selection patterns is, therefore, essential to avoid further promotion of the carbapenemase-resistant bacteria. However, this requires complete reconstruction of the mobile elements from the whole genome sequencing data, which has been notoriously hard due to their repetitive structure. Optical DNA mapping has recently been suggested as a novel method which can be used to validate plasmid assemblies by visualizing coarse-grained sequence information of large DNA molecules [13]. The method is particularly well suited for plasmids because sequence information can be obtained for single intact DNA molecules [14], and has previously been shown to be highly efficient in tracing resistance plasmids during outbreaks [15].

In this study, we aimed to characterize the resistomes of six highly multi-resistant pathogens – all phenotypically resistant to at least one carbapenem antibiotic – and the correspondence with their associated resistance phenotypes. To ensure completely reconstructed genome sequences, we used a combination of short- and long-read sequencing. Four novel resistance plasmids were identified and their complete assemblies were verified by optical DNA mapping. This enabled analysis of the full resistance genotype, including the acquired carbapenemase genes and their potential for horizontal gene transfer and co-selection by other classes of antibiotics. Careful manual comparison between the expected resistance phenotypes and the observed resistance levels showed a general agreement, but there were important discrepancies for four of the six isolates. Hence, our results further describe the genetic diversity of carbapenem resistance and underline the challenges with using WGS-based diagnostics for extensively drug-resistant bacteria.

## Methods

### Bacterial isolates

Clinical isolates showing reduced susceptibility to carbapenem antibiotics, when belonging to the genera *Pseudomonas* or *Acinetobacter* or the family *Enterobacteriaceae*, are routinely saved and stored at −80 °C at the Clinical Microbiology Laboratory at the Sahlgrenska University Hospital, Gothenburg, Sweden. The six strains analysed in the present study were isolated between 2012 and 2013, and were selected due to their multi-resistant phenotypes, including resistance to at least one carbapenem antibiotic. To enable analysis of resistance genes in different genetic backgrounds, isolates of different genera and species were chosen: *Escherichia coli, Klebsiella pneumoniae, Proteus mirabilis, Pseudomonas aeruginosa* and *Acinetobacter baumannii*. The strains originated from patients in the Gothenburg area, who were sampled either due to signs of infection or for screening purposes after hospital care abroad (Table S1, available in the online version of this article). Before storage, the strains were identified to the species level according to European guidelines for clinical laboratories (http://www.eucast.org/ast_of_bacteria/).

### DNA isolation

Bacterial DNA was obtained after plating the bacteria on blood agar medium and incubating overnight. For Illumina sequencing, DNA from bacterial cultures was isolated using a PureLink Genomic DNA kit (Invitrogen) according to the manufacturer’s description. For PacBio sequencing, DNA was isolated using the Genomic DNA Buffer Set and Genomic-tip 500/G (Qiagen). The quality and quantity of DNA were assessed using a NanoDrop ND 1000 spectrophotometer (Thermo Fisher), a Qubit 2.0 Fluorometer (Invitrogen) and visualization on an agarose gel before sending DNA for WGS. For the optical DNA mapping, plasmid DNA was prepared from an overnight culture with the Qiagen Plasmid Midi Kit according to the manufacturer’s description for low-copy plasmids.

### Whole genome sequencing

All isolates were index-tagged, pooled and sequenced at the Genomics Core Facility, University of Gothenburg, using Illumina MiSeq generating 250 bp paired reads. Trimming of adaptors and low-quality sequences was performed using Trim Galore! (www.bioinformatics.‌babraham.ac.uk/projects/trim_galore/) with default settings for paired-end data. The PacBio RS II system was used at the Science for Life Laboratory in Uppsala for long-read sequencing (fragment size 10–20 kb), utilizing one chip for each isolate. *De novo* hybrid assemblies using the quality controlled Illumina reads and the PacBio subreads were constructed using SPAdes v.3.7.0 (parameters –careful –cov-cutoff 10) [16]. The assembly graphs were used for additional manual scaffolding. Each resulting assembly (scaffolds >1 kb listed in Table 1) comprised one chromosome scaffold and one to six additional plasmid scaffolds.

**Table 1.** Assembled genomes and acquired antibiotic resistance genes

Strain	Scaffold ID	Length	Illumina coverage	Incompatibility group	MPF family*	MOB family†	Other T4SS genes	Acquired antibiotic resistance genes ordered including duplications‡
*Ps. aeruginosa* CCUG 70744	Chromosome	6.86 Mb	68×	–	–	–	–	–
*E. coli* CCUG 70745	Chromosome	5.07 Mb	75×	–	–	–		*bla*_CTX-M-15_#, *bla*_CMY-6_, *bla*_CMY-6_
	pEco70745_1	107 kb	86×	IncFIA, IncFIB, IncFII	F	F	*t4cp2*	*tet(B)*, *bla*_OXA-1_#, *aac(6′)-Ib-cr*#, [*dfrA17*, *ant(3″)-I*], *sul1, aph(6)-Id#*, *aph(3″)-Ib*, *aph(6)-Id*, *mph(A)#*, *erm(B)*
	pEco70745_2	44 kb	102×	IncX3	T	P	*t4cp1*, *t4cp2*, *virB4*	*bla*_NDM-7_#
	pEco70745_3	3.6 kb	828×	–	–	–	–	–
	pEco70745_4	1.9 kb	916×	–	–	–	–	–
	pEco70745_5	1.8 kb	704×	ColpVC	–	–	–	–
	pEco70745_6	1.5 kb	392×	–	–	–	–	–
*K. pneumoniae* CCUG 70742	Chromosome	5.26 Mb	69×	–	–	–	–	–
	pKpn70742_1	65 kb	191×	IncR	–	–	–	*dfrA14*, *tet(A)*, [*aac(6′)Ib-cr, bla*_OXA-1_], *bla*_CTX-M-15_, *bla*_TEM-1_, *aph(6)-Id*#, *aph(3″)-Ib*, *sul2*
	pKpn70742_2	63 kb	55×	IncL/M	I	P	*t4cp1*, *t4cp2*, *traU*	*bla*_OXA-48_#
	pKpn70742_3	4.4 kb	861×	–	–	–	–	–
	pKpn70742_4	1.9 kb	1093×	ColpVC	–	–	–	–
*Pr. mirabilis* CCUG 70746	Chromosome	3.91 Mb	103×	–	–	–	–	–
	pPmi70746_1	192 kb	189×	IncA/C2	F	H	*t4cp1*, *t4cp2*, *virB4*	*sul1*, [*ant(3″)-I*, *dfrA12*], *mph(E)*, *msr(E)*, *armA*, *sul1*, *bla*_NDM-1_#, *aph(3′)-VIa*, {1}, *sul1*, *bla*_NDM-1_#, *aph(3′)-VIa*, {1}, *sul1*, *bla*_NDM-1_#, *aph(3′)-VIa*, {1}, *sul1*, [*bla*_OXA-10_, *cmlA5*, *ARR-2*, *dfrA14*], *bla*_CMY-16_#, *sul2*, *aph(3″)-Ib*,*aph(6)-Id*, *tet(A)*, *floR*, {2}
*A. baumannii* CCUG 70743	Chromosome	3.94 Mb	111×	–	–	–	–	[*aac(3)-Ia*], *aph(3″)-Ib*, *aph(6)-Id*, *tet(B)*, *sul2*||
	pAba70743_1	11 kb	358×	–	–	–	–	*bla*_OXA-72_*, bla*_OXA-72_
	A5_NODE_3§	1.3 kb	81×	–	–	–	–	*aac(6′)-Im-*like
*K. pneumoniae* CCUG 70747	Chromosome	5.22 Mb	64×	–	–	–	–	–
	pKpn70747_1	186 kb	71×	IncFIB(K), IncFII	–	–	–	*sul1*, *aac(6′)-Ib*, *aph(3′)-Ic*#, [*dfrA1*, *ant(3″)-Ia*#], [*ant(3″)-Ia*#, *dfrA1*, *bla_VIM-1_*], *bla*_SHV-200_
	pKpn70747_2	118 kb	51×	IncFII(K)	F	F	*t4cp1*, *t4cp2*, *virB4*	*bla*_KPC-2_#, *bla*_TEM-1_
	pKpn70747_3	33 kb	350×	–	I	–	–	–

*Genes encoding the membrane-associated mating pair formation (MPF) complex which provides the mating channel for conjugative transfer.

†A set of mobility (MOB) genes essential for conjugative transfer including e.g. a relaxase.

‡Genes annotated in integron gene cassettes are marked by underlining and square brackets, [_]. The position of annotated ISCR elements and their type (1 or 2) are marked with a pair of curly brackets, {}.

§The assembly graph for *A. baumannii* CCUG 70743 and the best match against GenBank indicated that scaffold A5_NODE_3 was more likely to be a part of the chromosome than a plasmid.

||Genes annotated next to an insertion sequence (<200 bp in between) are marked with a hash.

### Optical DNA mapping

The sequence assemblies of the large plasmids were analysed using optical DNA mapping; for details about this method, see [14, 15]. To generate the barcode pattern – which is based on the underlying sequence – the DNA was stained before the nanofluidic experiments in the following way [17]. The extracted plasmids were mixed with YOYO-1 (ratio 1 : 5 with respect to DNA) and netropsin (ratio 150 : 1 with respect to YOYO-1) in Tris-borate-EDTA buffer. To reduce photo-nicking of the DNA, 2 % (v/v) b-mercaptoethanol was added to the solution. Nanofluidic chips were fabricated in oxidized silicon using standard methods described by Persson and Tegenfeldt [18]. The nanochannels used in the study had dimensions of 100×150 nm^2^ and a length of 500 µm. DNA was flushed into the microchannels to reach the nanochannel inlets and later forced into the nanochannels by using pressure-driven nitrogen flow. Plasmids in their circular form were inserted into the nanochannels and linearized using light irradiation. Two hundred images with 100 ms exposure time were obtained for each plasmid molecule using an EMCCD camera (Photometrics Evolve) in combination with an inverted fluorescence microscope (Zeiss AxioObserver.Z1) with a 100× oil immersion objective (NA=1.46). Theoretical optical DNA maps from assembled DNA sequences were created similar to Nilsson *et al*. [17] with updated ligand binding constants [19]. Comparisons between theoretical and experimental optical DNA maps were performed as described previously [14, 17].

### Genotype identification and annotation

The genomes of the following strains were used as references: *Ps. aeruginosa* PAO1 (RefSeq accession number NC_002516.2), *E. coli* str. K-12 substr. MG1655 (NC_000913.3), *K. pneumoniae* subsp. *pneumoniae* MGH 78578 (NC_009648.1), *Pr. mirabilis* HI4320 (NC_010554.1) and *A. baumannii* ATCC 17978 (NC_009085.1). Annotation of the genome assemblies and reference genomes was obtained using ResFinder with relaxed thresholds (%ID >40 % and minimum length >40 %) for mobile antibiotic resistance genes and PlasmidFinder with default thresholds for plasmid markers [20, 21]. To ensure high-quality annotation of mobile resistance genes, the ResFinder results were inspected manually to exclude incomplete genes and genes with nonsense mutations or frameshifts. Any low-quality hits were also studied manually to determine if they could be novel versions of known resistance genes. Any genes annotated in both the genome assemblies and their respective reference genome (Table S2) – such as chromosomal β-lactamases – were excluded so that only acquired genes remained for further analysis. CONJscan was used to detect genes involved in horizontal gene transfer with type IV secretion systems (T4SS) [22]. Chromosomal mutations were detected in selected genes (Table S3) by aligning each assembly against the corresponding reference gene using blast+ (tblastn, v2.2.30+, default parameters) [23]. All chromosomal reference sequences were collected from the reference genomes. Firstly, because chromosomal mutations in the quinolone target genes (*gyrAB*, *parCE*) is a common mechanism for clinical resistance against said antibiotics, all isolates were screened for mutations causing amino acid substitutions in these genes. Secondly, additional chromosomal genes were screened for mutations if there was a discrepancy between a measured phenotype and the expected phenotype based on the acquired mobile resistance genes. All genomes were, furthermore, annotated for putative integron-mediated gene cassettes. Putative *attC* sites were identified using HattCI (v1.0b, parameters -t -s 1000 t 6) [24] and had their secondary structure validated using Infernal (v1.1.1). The validation was based on a covariance model created from a structure-based alignment constructed from 109 manually curated *attC* sites using LocARNA (v1.8.9) [25, 26]. To complete the gene cassette annotation, genes were predicted upstream of the *attC* sites using Prodigal (v2.6.2). In addition, insertion sequences (ISs) in close proximity to resistance genes were identified using ISfinder [27]. Putative plasmid assemblies were screened against GenBank using blast+ (blastn, v2.2.30+, default parameters) to find the closest matching plasmid – defined as highest query coverage, thereafter highest identity – previously described. Next, plasmid assemblies were globally aligned against their closest GenBank match using Stretcher [28]. All matching plasmids were also annotated for antibiotic resistance genes and genes involved in T4SS, as described above. Finally, all regions encoding acquired carbapenemase genes were annotated for ISCR elements [29].

### Resistance phenotype identification

The MICs for the following antibiotics were measured for all isolates using Etest (bioMérieux) according to the manufacturer’s instructions: amikacin, gentamicin, tobramycin, ertapenem, imipenem, meropenem, cefotaxime, ceftazidime, ceftriaxone, cefuroxime, aztreonam, amoxicillin, amoxicillin/clavulanic acid, ampicillin, piperacillin/tazobactam, ciprofloxacin, levofloxacin, moxifloxacin, trimethoprim/sulfamethoxazole (co-trimoxazole), tetracycline, tigecycline, chloramphenicol, colistin, fosfomycin and rifampicin. The results were used to classify the isolates as either susceptible, intermediate or resistant to the tested antibiotics, based on EUCAST clinical breakpoints [30]. The measured antibiotic resistance phenotype was compared to the expected phenotype, which was obtained from the annotated genotype in the following way. For each annotated antibiotic resistance gene, the MICs associated with the antibiotics listed above were acquired from litterature for the relevant species. Based on these previously measured effects of the genes and EUCAST clinical breakpoints, each isolate was expected to be either susceptible, intermediate or resistant to the antibiotics of interest. This method for the prediction of resistance profiles has previously been used in several evaluations of WGS as a replacement for traditional susceptibility testing [31–34].

## Results

### Assemblies and mobility

**Fig. 1.**
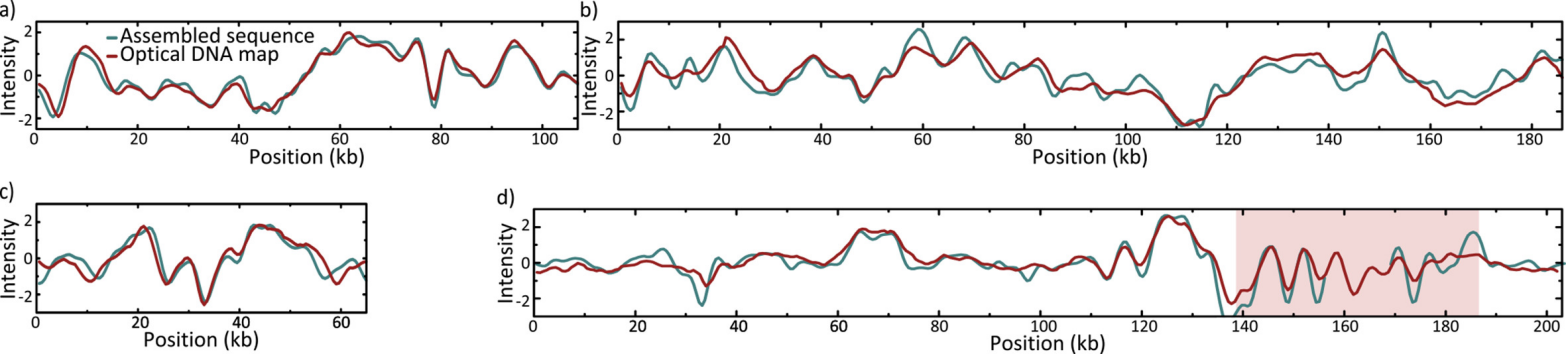
Comparison between the consensus of experimentally generated optical DNA maps (red) and theoretical optical DNA maps based on assembled sequences (teal) for novel plasmids (a) pEco70745_1 in *E. coli* CCUG 70745 (107 kb), (b) pKpn70747_1 in *K. pneumoniae* CCUG 70747 (186 kb), (c) pKpn70742_1 in *K. pneumoniae* CCUG 70742 (65 kb) and (d) pPmi70746_1 in *Pr. mirabilis* CCUG 70746 (experimental: 202 kb, theoretical: 192 kb). Intensity (*y*-axis) refers to the normalized emission intensity along the DNA molecule (*x*-axis), which correlates coarsely with the underlying DNA sequence. To aid the fitting of the barcodes in (d) – which are of different size – a gap has been introduced in the teal curve inside the red box. This box corresponds to a gene duplication and amplification region in pPmi70746_1 (see Fig. S1 for a histogram of individual plasmid lengths of pPmi70746_1).

**Fig. 2.**
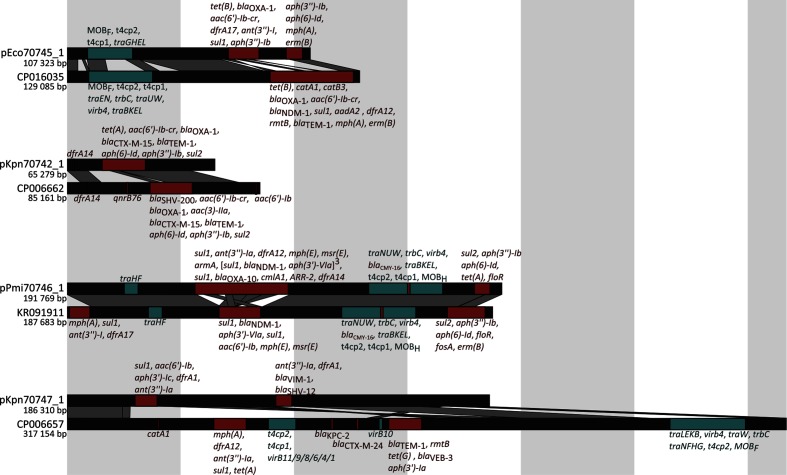
Comparisons between novel assembled plasmids and their closest match in GenBank. Red regions encode antibiotic resistance genes (identified using ResFinder), and teal regions encode genes involved in T4SS (identified using CONJscan). The respective genes of interest are given in order next to each region. Dark grey parallelogram represent blastn alignments of high identity (>99 %).

The hybrid assemblies using short- and long-read data produced high-quality complete genome sequences. Each assembly comprised one large chromosomal scaffold (3.91–6.86 Mb long) and, except for *Ps. aeruginosa* CCUG 70744, one to six additional plasmid scaffolds (1.6–192 kb long). The sizes and sequences of the larger assembled plasmids (≥44 kb) corresponded well with the experimentally determined optical DNA maps of intact linearized plasmids (Figs 1 and S1). The theoretical and experimental optical DNA maps of *Pr. mirabilis* CCUG 70746 plasmid deviated slightly from its optical DNA map due to a highly variable gene duplication and amplification region (red box in Figs 1d and S1d). Plasmid marker genes for ten incompatibility groups were detected on nine out of 15 plasmids (Table 1). In addition, genes involved in T4SS were annotated on six of the plasmids, including mobility genes (MOB) belonging to family C, F, H and P, as well as mating pair formation genes (MPF) from familiy F, I and P (Table 1). The isolates had acquired two to 20 different antibiotic resistance genes and mutations, and exhibited clinical resistance to a large number of antibiotics, including carbapenems (see Table S4 for MIC values). Four plasmids were novel and had either no close match in GenBank or a different set of antibiotic resistance genes compared to their closest match (Fig. 2 and Table S5). Seven integrons with one to four gene cassettes each were annotated in the studied genomes, where six integrons carried antibiotic resistance genes (Table 1) in their cassettes.

### Comparison of the antibiotic resistance genotype and phenotype

*Pseudomonas aeruginosa* CCUG 70744 was the only isolate without any acquired antibiotic resistance genes (Table 2). However, one amino acid substitution that has been previously associated with fluoroquinolone resistance was detected in GyrB [35], but none in GyrA, ParC or ParE. Because upregulation of efflux pumps and downregulation of porins are common mechanisms of resistance in *Ps. aeruginosa*, pumps known to be associated with antibiotic resistance and their regulatory proteins were analysed for alterations [36]. An insertion in the regulatory gene *mexZ* (399_400insG) was found, causing a frameshift and a premature stop codon which probably impairs the function of the gene. Missense mutations in *mexZ* have been previously linked to overexpression of the efflux pump MexXY and *inter alia* moderate resistance to amikacin [37]. Furthermore, the gene encoding the OprD porin had 15 amino acid substitutions in addition to alterations in nucleotides 1114–1149 causing ten amino acid substitutions and two deletions towards the C terminus. A similar version of the OprD protein – lacking only substitutions E202Q, E230K and G307D – has been described previously in an intermediate carbapenem-resistant *Ps. aeruginosa* isolate [38]. However, these mutations have also been observed also in susceptible isolates [39]. Single point mutations were detected in pumps MexCD-OprJ (MexC: S297A; MexD: E257Q, S845A; OprJ: M72V, Q270R) and MexXY (MexX: H119Y, K329Q, W358R; MexY: T543A); in PBPs PbpC (A104P), PbpG (S250N) and PonA (615_616insP); as well as in regulator proteins AmpD (A134V), MexT (F67I) and NalC (G71E). However, no mutations were found in the pumps MexAB-OprM or MexEF-OprN; PBPs DacB, DacC, FtsI, MrcB or PbpA; or regulator proteins CzcRS, MexR, NalD and NfxB. Therefore, the resistance against fluoroquinolones and amikacin acquired by *Ps. aeruginosa* CCUG 70744 was expected from the detected genotype, but not the high-level β-lactam resistance.

**Table 2.** Detected genotypes with expected and measured phenotypes of all strains

Strain	Antibiotic class	Genotype	Expected non-susceptible phenotypes*†	Measured non-susceptible phenotypes*†	No EUCAST breakpoint*‡	Discrepancies*§
*Ps. aeruginosa* CCUG 70744	Aminoglycosides	*mexZ* frameshift	AMK	AMK^I^	–	–
	β-lactams	–	AMC^iR^, AMP^iR^, AMX^iR^, CTX^iR^, CRO^iR^, CXM^iR^, ETP^iR^	AMC^iR^, AMP^iR^, AMX^iR^, ATM, CTX^iR^, CAZ, CRO^iR^, CXM^iR^, ETP^iR^, IPM, MEM, TZP	–	ATM, CAZ, IPM, MEM, TZP
	Fluoroquinolones	GyrB: S466F	CIP, LVX	CIP^I^, LVX	MXF	–
	Tetracyclines	–	TET^iR^, TGC^iR^	TET^iR^, TGC^iR^	–	–
	Co-trimoxazole	–	STX^iR^	STX^iR^	–	–
	Others	–	CHL^iR^, RIF^iR^	CHL^iR^, RIF^iR^	FOF	–
*E. coli* CCUG 70745	Aminoglycosides	*aac(6′)-Ib-cr, ant(3″)-Ia*, *aph(3″)-Ib, aph(6)-Id*	AMK, TOB	AMK^I^, TOB	–	–
	β-lactams	*bla_CMY-6_, bla_CTX-M-15_, bla_NDM-7_, bla_OXA-1_*	AMX, AMC, AMP, ATM, CTX, CAZ, CRO, CXM, ETP, IPM, MEM, TZP	AMX, AMC, AMP, ATM, CTX, CAZ, CRO, CXM, ETP, IPM, MEM, TZP	–	–
	Fluoroquinolones	GyrA: S83L, D87N ParC: S80I, A108V	CIP, LVX, MXF	CIP, LVX, MXF	–	–
	Macrolides	*erm(B)*, *mph(A)*	AZM^iR^, CLR^iR^, ERY^iR^	AZM^iR^, CLR^iR^, ERY^iR^	–	–
	Tetracyclines	*tet(B)*	TET	–	TET	–
	Co-trimoxazole	*dftA17*, *sul1*	SXT	SXT	–	–
	Others	–	RIF^iR^	CHL, RIF^iR^	–	CHL
*K. pneumoniae* CCUG 70742	Aminoglycosides	*aac(6′)-Ib-cr, aph(3″)-Ib, aph(6)-Id*	AMK, TOB	TOB	–	AMK
	β-lactams	*bla_CTX-M-15_, bla_OXA-1_, bla_OXA-48_, bla_TEM-1_*	AMX, AMC, AMP^iR^, ATM, CTX, CAZ, CRO, CXM, ETP, TZP	AMX, AMC, AMP^iR^, ATM, CTX, CAZ, CRO, CXM, ETP, TZP	–	–
	Fluoroquinolones	GyrA: Y83I; ParC: S80I	CIP, LVX, MXF	CIP, LVX^I^, MXF	–	–
	Macrolides	–	AZM^iR^, CLR^iR^, ERY^iR^	AZM^iR^, CLR^iR^, ERY^iR^	–	–
	Tetracyclines	*tet(A)*	TET	TGC	TET	TGC
	Co-trimoxazole	*dfrA14*, *sul2*	SXT	SXT	–	–
	Others	–	RIF^iR^	RIF^iR^	–	–
*Pr. mirabilis* CCUG 70746	Aminoglycosides	*ant(3″)-Ia*, *aph(3′)-VIa, aph(3″)-Ib*, *aph(6)-Id*, *armA*	AMK, GEN, TOB	AMK, GEN, TOB	–	–
	β-lactams	*bla*_CMY-16_, *bla*_NDM-1_, *bla*_OXA-10_	AMX, AMC, AMP, ATM, CTX, CAZ, CRO, CXM, ETP, IPM, MEM, TZP	AMX, AMC, AMP, ATM^I^, CTX, CAZ, CRO, CXM, ETP, IPM, MEM, TZP	–	–
	Fluoroquinolones	GyrA: S83I; ParC: S84I	CIP, LVX, MXF	CIP, LVX, MXF	–	–
	Macrolides	*mph(E)*, *msr(E)*	AZM^iR^, CLR^iR^, ERY^iR^	AZM^iR^, CLR^iR^, ERY^iR^	–	–
	Tetracyclines	*tet(A)*	TET^iR^, TGC^iR^	TET^iR^, TGC^iR^	–	–
	Co-trimoxazole	*sul1, sul2*, *dfrA12*, *dfrA14*	SXT	SXT	–	–
	Others	*aar-2*, *cmlA5, floR*	CHL, CST^iR^, RIF^iR^	CHL, CST^iR^, RIF^iR^	–	–
*A. baumannii* CCUG 70743	Aminoglycosides	*aac(3)-Ia,aac(6′)-Im*-like*, aph(3″)-Ib, aph(6)-Id*	AMK, GEN, TOB	AMK, GEN, TOB	–	–
	β-lactams	*bla*_OXA-72_	AMC^iR^, AMP^iR^, AMX^iR^, CTX^iR^, CRO^iR^, CXM^iR^, ETP^iR^, IPM, MEM	AMC^iR^, AMP^iR^, AMX^iR^, ATM^iR^, CTX^iR^, CRO^iR^, CXM^iR^, ETP^iR^, IPM, MEM	CAZ, TZP	–
	Fluoroquinolones	GyrA: S58L; ParC S84L	CIP, LVX	CIP, LVX	MXF	–
	Macrolides		AZM^iR^, CLR^iR^, ERY^iR^	AZM^iR^, CLR^iR^, ERY^iR^	–	–
	Tetracyclines	*tet(B)*	TET	–	TET, TGC	–
	Co-trimoxazole	*sul2*	SXT	SXT	–	–
	Others	–	FOF^iR^, RIF^iR^	FOF^iR^, RIF^iR^	CHL	–
*K. pneumoniae* CCUG 70747	Aminoglycosides	*aac(6′)-Ib*, *aac(6′)-Il*, *ant(3″)-Ia*, *aph(3′)-Ic*	AMK, TOB	AMK, TOB	–	–
	β-lactams	*bla*_KPC-2_, *bla*_TEM-1_*, bla*_VIM-1_	AMX, AMC, AMP^iR^, ATM, CTX, CAZ, CRO, CXM, ETP, IPM, MEM, TZP	AMX, AMC, AMP^iR^, ATM, CTX, CAZ, CRO, CXM, ETP, IPM, MEM, TZP	–	–
	Fluoroquinolones	GyrA: D87G; ParC: S80R	CIP, LVX, MXF	CIP, LVX, MXF	–	
	Tetracyclines	–	–	–	TET	–
	Co-trimoxazole	*dfrA1*, *sul1*	SXT	SXT	–	–
	Others	–	RIF^iR^	CHL, FOF, RIF^iR^	–	CHL, FOF

*AMK, amikacin; GEN, gentamicin; TOB, tobramycin; AMX, amoxicillin; AMC, amoxicillin/clavulanic acid; AMP, ampicillin; ATM, aztreonam; CTX, cefotaxime; CAZ, ceftazidime; CRO, ceftriaxone; CXM, cefuroxime; ETP, ertapenem; IPM, imipenem; MEM, meropenem; TZP, piperacillin/tazobactam; CIP, ciprofloxacin; LVX, levofloxacin; MXF, moxifloxacin; AZM, azithromycin; CLR, clarithromycin; ERY, erythromycin; TET, tetracycline; TGC, tigecycline; SXT, trimethoprim/sulfamethoxazole; CHL, chloramphenicol; CST, colistin; FOF, fosfomycin; and RIF, rifampicin.

†No superscript for resistant, I for intermediate and iR for intrinsically resistant, as defined by EUCAST [30, 111].

‡No clinical breakpoints to compare MIC with and not listed as intrinsically resistant.

§Any differences between expected and measured phenotype, excluding antibiotics lacking EUCAST clinical breakpoints.

In *E. coli* CCUG 70745, four aminoglycoside-modifying enzymes were detected: an acetyltransferase also able to acetylate the fluoroquinolones ciprofloxacin and norfloxacin (*aac(6′)-Ib-cr*) [40]; a nucleotidyltransferase (*ant(3″)-Ia*) [41]; and two phosphotransferases (*aph(3″)-Ib*, *aph(6)-Id*) [42]. The isolate also carried four β-lactamases (*bla*_CMY-6_, *bla*_CTX-M-15_, *bla*_NDM-7_, *bla*_OXA-1_) which are more than sufficient to hydrolyse all known β-lactams [43–45]. The *bla*_NDM-7_ gene was flanked upstream by an IS5 element and a truncated ISAba125. Furthermore, *E. coli* CCUG 70745 had acquired a *tet(B)* efflux pump that has previously been linked to tetracycline but not tigecycline resistance, a macrolide phosphorylase (*mph(A)*) and an rRNA methylase (*erm(B)*) [46–49]. Two genes maintaining the folate synthesis targeted by trimethoprim (*drfA17*) and sulfamethoxazole (*sul2*) were also found [50, 51]. Lastly, the following fluoroquinolone resistance mutations in the target genes were detected: S83L and D87N in GyrA; and S80I and A108V in ParC [52]. The only observed antibiotic phenotype that was not expected in *E. coli* CCUG 70745 was chloramphenicol resistance. There was, however, a gene similar to phenicol-inactivating enzymes, which could potentially be a novel, previously uncharacterized, phenicol resistance gene (Fig. S2). Although located in the chromosome, it was not present in the reference genome *E. coli* K-12 substr. MG1655. Phylogenetic analysis showed that the novel gene was most closely related to the type B phenicol acetyltransferases, CatB (Fig. S2a), with the closest match being of type B-5, CatB9 (31.6 % global identity) (Fig. S2c) [53]. The sequence was, however, less similar to the CatB9 sequence than the closely clustered CatB group, and could, therefore, constitute a novel type of Cat enzymes. The sequence is 100 % identical to GenBank multispecies entry WP_020232949.1 annotated in 26 *E. coli* genomes and one *K. pneumoniae*. However, the function of this gene has so far not been verified experimentally. In proximity to the putative gene, there were two ISs: one belonging to family IS4 (99.6 % identical to IS4) 9.5 kb downstream, and one to IS1 (95.4 % identical to IS1X2) 1.3 kb downstream.

The *K. pneumoniae* CCUG 70742 isolate had acquired three aminoglycoside resistance genes: the same acetyltransferase (*aac(6')-Ib-cr*) and phosphotransferases (*aph(3'')-Ib*, *aph(6)-Id*) as *E. coli* CCUG 70745 [40, 51]. Four β-lactamases were also present (*bla*_CTX-M-15_, *bla*_OXA-1_, *bla*_OXA-48_, *bla*_TEM-1_) and were expected to provide a clinical level of resistance towards all tested β-lactams except the carbapenems imipenem and meropenem [43, 45, 54–57]. The *bla*_OXA-48_ gene was flanked downstream by an IS10A element. Two genes protecting against folate synthesis inhibitors (*sul2*, *dftA14*) were detected, as were two substitutions in GyrA (Y83I) and ParC (S80I) that have previously been associated with fluoroquinolone resistance [51, 58, 59]. The acquired efflux pump *tet(A)* has been linked to tetracycline but not tigecycline resistance [60]. Because upregulation of chromosomally encoded efflux pumps is a known mechanism of tigecycline resistance in *K. pneumoniae*, a number of chromosomal genes were analysed for mutations [61, 62]. Single substitutions were detected in the global regulator protein MarR (S3N) and outer membrane protein TolC (T480N), but regulators AcrR, MarA, RamAR and SoxRS did not have any alterations, nor did efflux pump AcrAB. However, none of the detected alterations has previously been linked to tigecycline resistance. Altogether, the phenotypic resistance profile of *K. pneumoniae* CCUG 70742 was expected from the genotype, except the lack of amikacin resistance and the acquired tigecycline resistance.

In *Pr. mirabilis* CCUG 70746, the detected 16S methyltransferase (*armA*) provides resistance against all aminoglycosides tested in this study [63]. However, the isolate carried four additional aminoglycoside-modifying enzymes: one nucleotidyltransferase (*ant(3″)-Ia*-like) and three phosphotransferases (*aph(3′)-VIa*-like, *aph(3″)-Ib*, *aph(6)-Id*) [42, 64, 65]. The three detected β-lactamases (*bla*_CMY-16_, *bla*_NDM-1_, *bla*_OXA-10_) are able to hydrolyse all tested β-lactams [66–68]. Furthermore, the isolate had obtained a tetracycline efflux pump (*tet(A)*), a rifampicin ADP-ribosylating transferase (*arr-2*), a macrolide phosphorylase (*mph(E)*), as well as an erythromycin and streptogramin B efflux pump (*msr(E)*) [69–71]. Two sulphonamide (*sul1*, *sul2*), two trimethoprim (*dfrA12*, *dfrA14*-like) and two phenicol (*cmlA5*, *floR*-like) resistance genes were also present [51, 58, 72–76]. Lastly, fluoroquinolone resistance mutations were detected in both GyrA (S83I) and ParC (S84I) [77]. In total, *Pr. mirabilis* CCUG 70746 had acquired 18 different antibiotic resistance genes making it non-susceptible to all tested antibiotics except for fosfomycin. This antibiotic resistance profile concurred with what was expected from the acquired genes and mutations. Interestingly, optical DNA mapping demonstrated varying sizes of plasmid pPmi70746_1, which we were able to reveal due to the single molecule nature of the method (Fig. S1d). The range in sizes was due to differences in a region where a 6.5 kb long multi-copy region was annotated. This region was present in three copies in the assembly and encoded antibiotic resistance genes *bla*_NDM-1_, *aph(3′)-VIa* and *sul1* (Fig. 1d). There were at least three different versions of the plasmid observed, but due to the large variability in the sample and the uncertainty in the length estimation, we were unable to confidently determine the exact number. Four IS common region (ISCR1) transposases were found before and after each entry of the multi-copy region. Moreover, all copies of the *bla*_NDM-1_ gene were flanked downstream by ISAba125, and an ISCR2 transposase was annotated outside of the multi-copy region, close downstream to the *floR* gene.

The strain *A. baumannii* CCUG 70743 had acquired four different aminoglycoside-modifying enzymes: two acetyltransferases (*aac(3)-Ia*, *aac(6′)-Im*-like) and two phosphotransferases (*aph(3″)-Ib*, *aph(6)-Id*) [42, 78, 79]. It had also obtained one carbapenemase (*bla*_OXA-72_) [80] and a tetracycline efflux pump (*tet(B)*). Because *A. baumannii* is considered intrinsically resistant to trimethoprim, the detected sulphonamide resistance gene (*sul2*) was expected to be sufficient to gain co-trimoxazole resistance. The alterations in GyrA (S58L) and ParC (284L) have been detected previously together in fluoroquinolone-resistant *A. baumannii* isolates [81]. Therefore, all acquired resistance in *A. baumannii* CCUG 70743 concurred with the expected pattern from its genotype.

The last strain, *K. pneumoniae* CCUG 70747, encoded multiple aminoglycoside resistance genes: two acetyltransferases (*aac(6′)-Ib*, *aac(6′)-Il*), one nucleotidyltransferase (*ant(3″)-Ia*) and one phosphotransferase (*aph(3′)-Ic* [82]) [41, 83]. In addition, it had acquired four β-lactamases (*bla*_KPC-2_, *bla*_SHV-200_, *bla*_TEM-1_, *bla*_VIM-1_) that are expected to hydrolyse all tested β-lactams [57, 84, 85]. This is the first time the gene *bla*_SHV-200_ has been described (accession number MF871643). The gene *bla*_KPC-2_ was flanked upstream by ISKpn7 and downstream by ISKpn6. The plasmid encoding *bla*_VIM-1_ was novel and did not have high identity to any other plasmid in GenBank. Furthermore, the carbapenemase *bla*_VIM-1_ was found in an integron together with a truncated *aac(6′)-Id*, and complete *dfrA1* and *ant(3'')-Ia* genes. Each gene was followed by an *attC* site, and the same integron cassette has previously been detected in *E. coli*, *Pr. mirabilis* and *Providencia stuartii* [86]. The isolate had also acquired sulphonamide and trimethoprim resistance genes (*dfrA1*, *sul1*) that together provide resistance against co-trimoxazole [72, 87]. The alterations detected in GyrA (D87G) and ParC (S80R) have previously been detected separately in fluoroquinolone-resistant *K. pneumoniae* [88], but this particular combination of mutations has not been characterized before. It was, therefore, non-trivial to predict the corresponding resistance phenotype, but based on the precautionary principle it could be assumed that the combination would cause a clinical level of resistance. Therefore, all resistance phenotypes agreed with the expected resistance pattern, except for the chloramphenicol resistance.

## Discussion

In this study, we have determined the resistomes of six carbapenem-resistant clinical isolates of five different gram-negative species. All isolates had acquired a vast arsenal of mobile antibiotic resistance genes and chromosomal mutations, except for *Ps. aeruginosa* CCUG 70744. The *A. baumannii* and *Enterobacteriaceae* isolates encoded 7–18 different resistance genes, some of which were annotated in multiple copies within the same isolate. The correspondence between the measured resistance profiles and the expected profile – based on acquired genes and mutations – was high, but with significant exceptions. The combination of WGS using Illumina and PacBio sequencing with optical DNA mapping produced reliable high-quality assemblies which enabled us to study the genetic context of the carbapenemase genes, and determine which of the resistance genes were present on the same plasmids. Indeed, four novel putative plasmids were fully assembled, of which one encoded a carbapenemase. In addition, the use of optical DNA maps revealed a multi-copy region of the plasmid carrying the *bla*_NDM-1_ gene in *Pr. mirabilis* CCUG 70746. Several versions of the plasmid pPmi70746_1 – with a different number of copies of this region – were harboured by a population obtained from a non-selective medium. Because most genome assembly algorithms do not take into account the presence of different clones within the sequenced material, this feature would have been easily overlooked without the use of single molecule techniques. It has been shown previously that gene duplication and amplification is a mechanism for bacteria to increase the expression of antibiotic resistance factors, including the gene *bla*_NDM-1_ [89, 90]. In general, failing to identify this specific genetic context of resistance genes may therefore result in incorrect predictions of the resistance phenotypes.

Six different carbapenemase genes had been acquired among the isolates, belonging to functional groups 2df (*bla*_OXA-48_, *bla*_OXA-72_), 2f (*bla*_KPC-2_) and 3a (*bla*_NDM-1_, *bla*_NDM-7_, *bla*_VIM-1_) [6]. In addition, two intrinsic carbapenemase genes were detected in the chromosomes, both belonging to functional groups 2df and molecular class D: *bla*_OXA-50_-like (amino acid substitutions T16A and V148A) in *Ps. aeruginosa* CCUG 70744 and *bla*_OXA-66_ in *A. baumannii* CCUG 70743 [91]. The *bla*_OXA-50_*-*like group of enzymes (also known as PoxB) hydrolyse imipenem only at a slow rate and have a weak affinity to meropenem [92], and *bla*_OXA-66_ does not provide clinical levels of carbapenem resistance even when overexpressed [93]. This – together with the lack of acquired carbapenemase genes in *Ps. aeruginosa* CCUG 70744 – shows the large diversity of the genetic basis of carbapenem resistance among the studied strains. All six acquired carbapenemases were plasmid-borne, where four of these plasmids also carried genes involved in T4SS [94], suggesting that they have the potential for conjugative self-transfer. While most of the resistance plasmids carried multiple resistance genes for different antibiotic classes, *bla*_NDM-7_, *bla*_OXA-48_ and *bla*_OXA-72_ were the only annotated resistance genes on their respective plasmids, and *bla_KPC-2_* was only joined by one additional resistance gene (*bla*_TEM-1_). Similarly, a single plasmid with no additional resistance genes has been linked to the majority of the dissemination of *bla*_OXA-48_ in Europe and the Middle East [95] and, to the best of our knowledge, neither *bla*_OXA-72_ nor *bla*_NDM-7_ has yet been annotated together with other resistance genes. Carbapenems were introduced to the market after cephalosporins and penicillins, and carbapenemase genes may therefore not yet had the time to be incorporated into existing resistance plasmids. Nevertheless, it implies a limited risk for co-selection due to genetic co-localization of these carbapenemase genes with other classes of antibiotics. However, other mechanisms of co-selection – such as co-regulation, where one resistance factor is transcriptionally linked to another – cannot be excluded [96]. On the other hand, *bla*_NDM-1_ has been annotated together with several different resistance genes [97] and, in *Pr. mirabilis* CCUG 70746, it was co-located with resistance genes active against aminoglycosides, macrolides, lincosamides, streptogramin, phenicols, rifampicin, sulphonamides, tetracyclines and trimethoprim. Furthermore, *bla*_VIM-1_ was co-located with genes active against aminoglycosides, sulphonamides and trimethoprim. For these plasmids, there is a risk of creating a selective advantage for these carbapenemase genes by treating with, for example, co-trimoxazole.

Four of the isolates (*E. coli* CCUG 70745, *K. pneumoniae* CCUG 70742, *Pr. mirabilis* CCUG 70746 and *K. pneumoniae* CCUG 70747) had acquired three or four different β-lactamases, some of which were found in several copies. For example, *Pr. mirabilis* CCUG 70746 carried three different β-lactamases (*bla*_CMY-16_, *bla*_NDM-1_, *bla*_OXA-10_) within a single plasmid, where only *bla*_NDM-1_ would have been sufficient to hydrolyse all known β-lactams, except monobactams [67]. Why bacteria arm themselves with this redundancy of resistance genes is not clear. There could be a selective advantage of having a combination of β-lactamases of different spectra if they somehow complement each other, for example due to complementing hydrolysing efficiency for different β-lactams. It is also possible that only a subset of the β-lactamases is expressed in these isolates, as has been observed previously [98]. This would also suggest that the process of incorporating additional genes with wider spectra is, in general, quicker than the process of losing genes. Reversal of resistance, even in the absence of a selection pressure, has indeed been suggested to be slow or even non-existent [99]. Further studies of gene expression in bacteria encoding multiple β-lactamases are therefore needed to elucidate the reasons behind this genetic redundancy.

The correspondence between measured antibiotic resistance and the phenotypes expected from the annotated resistome was high, but there were important discrepancies. Complete agreement between the measured and expected resistance profile was observed only for two of the isolates: *Pr. mirabilis* CCUG 70746 and *A. baumannii* CCUG 70743. The expectations for co-trimoxazole were accurate for all isolates and resistance phenotypes were caused by acquired *sul* and *dfr* genes. For fosfomycin, the measured phenotypes were in agreement with what was expected, except for *K. pneumoniae* CCUG 70747 which was fosfomycin-resistant. This phenotype could potentially be caused by upregulation of the *fosA* gene, which was annotated in both studied *K. pneumoniae* strains, but excluded from our analysis due to its presence in the reference genome. It should be noted that the wild-type distribution of fosfomycin MICs for *K. pneumoniae* covers the clinical breakpoint (MIC>32 mg l^−1^), but it is not listed as intrinsically fosfomycin-resistant by EUCAST [100].

Correct predictions were also made for β-lactam resistance – which could be linked to acquired β-lactamases – for all isolates, except *Ps. aeruginosa* CCUG 70744. Altered gene expression is a common mechanism of resistance in *Ps. aeruginosa* and could putatively explain the acquired β-lactam resistance in strain CCUG 70744. Frameshift mutations such as the detected insertion interrupting the *mexZ* gene have previously been associated with upregulation of the MexXY-OprM pump and clinical level of resistance against amikacin [37]. Although this pump system has also been shown to extrude certain β-lactams, including carbapenems, upregulation has been shown not to be sufficient to reach clinical resistance [101]. Furthermore, loss of OprD expression is the most common mechanism of clinical carbapenem resistance in Europe [102], and there was a 12 amino acid long substitution detected in the OprD porin and a substitution in MexT which negatively regulates OprD expression [36]. Neither these alterations nor those detected in MexCDXY, OprJ, AmpD, NalC, PbpCG or PonA has previously been associated with antibiotic resistance, but we cannot exclude the possibility that they – singly or in combination with other genetic alterations – could contribute to the resistance phenotype. Similarly, altered gene expression of, for example, efflux pumps is a known tigecycline resistance mechanism in *K. pneumoniae* [61, 62]. Four of the isolates – *E. coli* CCUG 70745, *K. pneumoniae* CCUG 70742, *Pr. mirabilis* CCUG 70746 and *A. baumannii* CCUG 70743 – had acquired tetracycline efflux pumps (*tet*) that are expected to cause resistance against tetracycline but not tigecycline. This pattern was observed in *E. coli* CCUG 70745 but not in *K. pneumoniae* CCUG 70742, which was phenotypically tigecycline-resistant. Although no mutations previously linked to tigecycline resistance were found, it is still possible that altered gene expression was the underlying resistance mechanism in *K. pneumoniae* CCUG 70742. This implies that prediction of overexpressed intrinsic resistance genes directly from genomic data is challenging and requires additional analysis using, for example, gene expression assays.

Mutations in chromosomal non-mobile DNA can have a significant impact on the resistance phenotype and it is therefore essential to include point mutations in any attempt to fully describe the resistome. Indeed, all isolates included in this study had acquired fluoroquinolone resistance, which could be linked to known amino acid substitutions in the enzymes targeted by fluoroquinolones. The combination of amino acid substitutions detected in *K. pneumoniae* CCUG 70747 (D87G in GyrA and S80R in ParC) has not been previously described but resulted in high levels of fluoroquinolone resistance. This demonstrates that novel combinations of resistance mutations appear in clinical isolates and that further characterization of combinations of chromosomal mutations is necessary to ensure correct prediction of their corresponding resistance phenotype.

Regarding chloramphenicol, *Pr. mirabilis* CCUG 70746 had acquired two phenicol resistance genes (*cmlA4* and *floR*) and was indeed resistant. However, two additional isolates (*E. coli* CCUG 70745 and *K. pneumoniae* CCUG 70747) were chloramphenicol-resistant with no known genetic mechanism for phenicol resistance. In *E. coli* CCUG 70745, a gene encoding a putative novel phenicol-inactivating enzyme was, however, detected (Fig. S2b), showing low homology to known chloramphenicol resistance genes. The results highlight the importance of having complete databases with associated phenotypes when attempting to predict the susceptibility profile of an isolate. However, as new resistance mechanisms being mobilized into pathogenic bacteria is an ongoing evolutionary process, the databases will, in theory, never be complete. Nevertheless, continued efforts are necessary to keep databases as up to date as possible. This, in combination with systematic experimental validation of novel genes appearing in clinical isolates, is necessary to ensure a correct derived phenotype induced by resistance genes that have recently been mobilized into pathogens.

The predictions regarding aminoglycoside resistance were correct for all strains except *K. pneumoniae* CCUG 70742, which was expected to be amikacin-resistant. *K. pneumoniae* CCUG 70742 carried the gene *aac(6′)-Ib-cr*, which is active against amikacin, although less effective than the related gene *aac(6′)-Ib* [40]. However, identical *aac(6′)-Ib-cr* genes were found in both *E. coli* CCUG 70745 and *K. pneumoniae* CCUG 70742, where the *E. coli* strain showed intermediate resistance to amikacin while *K. pneumoniae* CCUG 70742 was classified as susceptible. This indicates that the genetic context of genes, and not just their presence, may be relevant in predicting the resistance profile. Indeed, it has been shown that the broad-spectrum β-lactamase gene *bla*_NDM-1_ gives different resistance profiles in different bacterial species [103].

In conclusion, the combination of short- and long-read sequencing with optical DNA mapping proved most useful to characterize the genome of six carbapenem-resistant clinical isolates. The resulting complete assemblies included four novel plasmids, one of which encoded a carbapenemase. This methodology enabled assessment of the risk for co-selection and spread of the annotated resistance genes. Furthermore, the concordance between the resistance level expected from the genotypes and the measured phenotype was overall good. Although the number of studied isolates was limited, we encountered many of the complexities of predicting a resistance profile from whole genome data. Firstly, resistance caused by altered expression of intrinsic genes – which is an important mechanism for clinical resistance against a wide range of antibiotic classes [61, 104, 105] – is inherently difficult to predict from genomic data. Secondly, if multiple resistance factors with low efficiency have been acquired by an isolate, it is not trivial to predict their combined effect. For example, ertapenem resistance has been observed in *E. coli* and *K. pneumoniae* lacking carbapenemases through a combination of extended-spectrum β-lactamases and loss of porin expression [106]. These mechanisms of resistance are typically not able to cause carbapenem resistance on their own. However, systematic studies of the combinatorial effects of multiple resistance genes, mutations and other genetic alterations are still very scarce and – due to their often complex interactions – the corresponding phenotype is hard to predict. Thirdly, different genetic contexts of one gene may alter its associated resistance phenotype [107, 108]. Lastly, the completeness of the database of mobile resistance genes can also affect the predicted phenotype if a novel resistance gene is present. This happened, for example, in 2016 when the mobile colistin resistance gene *mcr-1* was discovered in China, and quickly thereafter was annotated in genomes and metagenomes already present in public sequence databases [109, 110]. This emphasizes the importance in continuing the description of the genetic mechanisms behind antibiotic-resistant bacteria and their associated phenotypes. Our results therefore support the concerns raised in the EUCAST report on using WGS for antimicrobial susceptibility testing of bacteria [11]. While some of these issues might be addressed through more research – such as increasing the completeness of the databases through the discovery of novel genes – it is unlikely that the level of conferred resistance caused by altered expression of intrinsic genes will ever be correctly predicted from genomic data. Even though WGS will probably prove to be highly valuable in bacterial diagnostics, our results show that there will be many situations where the method will fall short due to its technical limitations.

## Supplementary Data

Supplementary File 1

## References

[1] Potter RF, D'Souza AW, Dantas G (2016). The rapid spread of carbapenem-resistant Enterobacteriaceae. Drug Resist Updat.

[2] World Health Organization (2017). Global priority list of antibiotic-resistant bacteria to guide research, discovery, and development of new antibiotics.

[3] U.S Department of Health and Human Services, Center for Disease Control and Prevention (2013). Antibiotic resistance threats in the United States.

[4] European Centre for Disease Prevention and Control (2017). Antimicrobial resistance surveillance in Europe 2015. Annual Report of the European Antimicrobial Resistance Surveillance Network (EARS-Net).

[5] Papp-Wallace KM, Endimiani A, Taracila MA, Bonomo RA (2011). Carbapenems: past, present, and future. Antimicrob Agents Chemother.

[6] Bush K, Jacoby GA (2010). Updated functional classification of beta-lactamases. Antimicrob Agents Chemother.

[7] Cerqueira GC, Earl AM, Ernst CM, Grad YH, Dekker JP (2017). Multi-institute analysis of carbapenem resistance reveals remarkable diversity, unexplained mechanisms, and limited clonal outbreaks. Proc Natl Acad Sci USA.

[8] Berglund F, Marathe NP, Österlund T, Bengtsson-Palme J, Kotsakis S (2017). Identification of 76 novel B1 metallo-β-lactamases through large-scale screening of genomic and metagenomic data. Microbiome.

[9] Mathers AJ, Peirano G, Pitout JD (2015). The role of epidemic resistance plasmids and international high-risk clones in the spread of multidrug-resistant Enterobacteriaceae. Clin Microbiol Rev.

[10] Carattoli A (2013). Plasmids and the spread of resistance. Int J Med Microbiol.

[11] Ellington MJ, Ekelund O, Aarestrup FM, Canton R, Doumith M (2017). The role of whole genome sequencing in antimicrobial susceptibility testing of bacteria: report from the EUCAST subcommittee. Clin Microbiol Infect.

[12] Köser CU, Ellington MJ, Peacock SJ (2014). Whole-genome sequencing to control antimicrobial resistance. Trends Genet.

[13] Müller V, Westerlund F (2017). Optical DNA mapping in nanofluidic devices: principles and applications. Lab Chip.

[14] Nyberg LK, Quaderi S, Emilsson G, Karami N, Lagerstedt E (2016). Rapid identification of intact bacterial resistance plasmids via optical mapping of single DNA molecules. Sci Rep.

[15] Müller V, Karami N, Nyberg LK, Pichler C, Torche Pedreschi PC (2016). Rapid tracing of resistance plasmids in a nosocomial outbreak using optical DNA mapping. ACS Infect Dis.

[16] Bankevich A, Nurk S, Antipov D, Gurevich AA, Dvorkin M (2012). SPAdes: a new genome assembly algorithm and its applications to single-cell sequencing. J Comput Biol.

[17] Nilsson AN, Emilsson G, Nyberg LK, Noble C, Stadler LS (2014). Competitive binding-based optical DNA mapping for fast identification of bacteria–multi-ligand transfer matrix theory and experimental applications on *Escherichia coli*. Nucleic Acids Res.

[18] Persson F, Tegenfeldt JO (2010). DNA in nanochannels—directly visualizing genomic information. Chem Soc Rev.

[19] Dvirnas A, Pichler C, Stewart CL, Quaderi S, Nyberg LK (2018). Facilitated sequence assembly using densely labeled optical DNA barcodes: A combinatorial auction approach. PLoS One.

[20] Carattoli A, Zankari E, García-Fernández A, Voldby Larsen M, Lund O (2014). *In silico* detection and typing of plasmids using PlasmidFinder and plasmid multilocus sequence typing. Antimicrob Agents Chemother.

[21] Zankari E, Hasman H, Cosentino S, Vestergaard M, Rasmussen S (2012). Identification of acquired antimicrobial resistance genes. J Antimicrob Chemother.

[22] Guglielmini J, de La Cruz F, Rocha EP (2013). Evolution of conjugation and type IV secretion systems. Mol Biol Evol.

[23] Camacho C, Coulouris G, Avagyan V, Ma N, Papadopoulos J (2009). BLAST+: architecture and applications. BMC Bioinformatics.

[24] Pereira MB, Wallroth M, Kristiansson E, Axelson-Fisk M (2016). HattCI: fast and accurate *attC* site identification using hidden Markov models. J Comput Biol.

[25] Nawrocki EP, Eddy SR (2013). Infernal 1.1: 100-fold faster RNA homology searches. Bioinformatics.

[26] Will S, Joshi T, Hofacker IL, Stadler PF, Backofen R (2012). LocARNA-P: accurate boundary prediction and improved detection of structural RNAs. RNA.

[27] Siguier P, Perochon J, Lestrade L, Mahillon J, Chandler M (2006). ISfinder: the reference centre for bacterial insertion sequences. Nucleic Acids Res.

[28] Rice P, Longden I, Bleasby A (2000). EMBOSS: the European molecular biology open software suite. Trends Genet.

[29] Toleman MA, Bennett PM, Walsh TR (2006). ISCR elements: novel gene-capturing systems of the 21st century?. Microbiol Mol Biol Rev.

[30] EUCAST (2016). Breakpoint tables for interpretation of MICs and zone diameters. Version 6.0. The European Committee on Antimicrobial Susceptibility Testing.

[31] Zankari E, Hasman H, Kaas RS, Seyfarth AM, Agersø Y (2013). Genotyping using whole-genome sequencing is a realistic alternative to surveillance based on phenotypic antimicrobial susceptibility testing. J Antimicrob Chemother.

[32] Stoesser N, Batty EM, Eyre DW, Morgan M, Wyllie DH (2013). Predicting antimicrobial susceptibilities for *Escherichia coli* and *Klebsiella pneumoniae* isolates using whole genomic sequence data. J Antimicrob Chemother.

[33] Bradley P, Gordon NC, Walker TM, Dunn L, Heys S (2015). Rapid antibiotic-resistance predictions from genome sequence data for *Staphylococcus aureus* and *Mycobacterium tuberculosis*. Nat Commun.

[34] Zhao S, Tyson GH, Chen Y, Li C, Mukherjee S (2016). Whole-genome sequencing analysis accurately predicts antimicrobial resistance phenotypes in *Campylobacter* spp. Appl Environ Microbiol.

[35] Mouneimné H, Robert J, Jarlier V, Cambau E (1999). Type II topoisomerase mutations in ciprofloxacin-resistant strains of *Pseudomonas aeruginosa*. Antimicrob Agents Chemother.

[36] Lister PD, Wolter DJ, Hanson ND (2009). Antibacterial-resistant *Pseudomonas aeruginosa*: clinical impact and complex regulation of chromosomally encoded resistance mechanisms. Clin Microbiol Rev.

[37] Vogne C, Aires JR, Bailly C, Hocquet D, Plésiat P (2004). Role of the multidrug efflux system MexXY in the emergence of moderate resistance to aminoglycosides among *Pseudomonas aeruginosa* isolates from patients with cystic fibrosis. Antimicrob Agents Chemother.

[38] El Amin N, Giske CG, Jalal S, Keijser B, Kronvall G (2005). Carbapenem resistance mechanisms in *Pseudomonas aeruginosa*: alterations of porin OprD and efflux proteins do not fully explain resistance patterns observed in clinical isolates. APMIS.

[39] Pirnay JP, de Vos D, Mossialos D, Vanderkelen A, Cornelis P (2002). Analysis of the *Pseudomonas aeruginosa oprD* gene from clinical and environmental isolates. Environ Microbiol.

[40] Robicsek A, Strahilevitz J, Jacoby GA, Macielag M, Abbanat D (2006). Fluoroquinolone-modifying enzyme: a new adaptation of a common aminoglycoside acetyltransferase. Nat Med.

[41] Sandvang D (1999). Novel streptomycin and spectinomycin resistance gene as a gene cassette within a class 1 integron isolated from *Escherichia coli*. Antimicrob Agents Chemother.

[42] Scholz P, Haring V, Wittmann-Liebold B, Ashman K, Bagdasarian M (1989). Complete nucleotide sequence and gene organization of the broad-host-range plasmid RSF1010. Gene.

[43] Karim A, Poirel L, Nagarajan S, Nordmann P (2001). Plasmid-mediated extended-spectrum β-lactamase (CTX-M-3 like) from India and gene association with insertion sequence ISEcp1. Fems Microbiol Lett.

[44] Göttig S, Hamprecht AG, Christ S, Kempf VA, Wichelhaus TA (2013). Detection of NDM-7 in Germany, a new variant of the New Delhi metallo-β-lactamase with increased carbapenemase activity. J Antimicrob Chemother.

[45] Ouellette M, Bissonnette L, Roy PH (1987). Precise insertion of antibiotic resistance determinants into Tn21-like transposons: nucleotide sequence of the OXA-1 beta-lactamase gene. Proc Natl Acad Sci USA.

[46] Lawley TD, Burland V, Taylor DE (2000). Analysis of the complete nucleotide sequence of the tetracycline-resistance transposon Tn10. Plasmid.

[47] Hirata T, Saito A, Nishino K, Tamura N, Yamaguchi A (2004). Effects of efflux transporter genes on susceptibility of *Escherichia coli* to tigecycline (GAR-936). Antimicrob Agents Chemother.

[48] Noguchi N, Emura A, Matsuyama H, O'Hara K, Sasatsu M (1995). Nucleotide sequence and characterization of erythromycin resistance determinant that encodes macrolide 2'-phosphotransferase I in *Escherichia coli*. Antimicrob Agents Chemother.

[49] Billard-Pomares T, Tenaillon O, Le Nagard H, Rouy Z, Cruveiller S (2011). Complete nucleotide sequence of plasmid pTN48, encoding the CTX-M-14 extended-spectrum β-lactamase from an *Escherichia coli* O102-ST405 strain. Antimicrob Agents Chemother.

[50] White PA, McIver CJ, Deng Y, Rawlinson WD (2000). Characterisation of two new gene cassettes, *aadA5* and *dfrA17*. FEMS Microbiol Lett.

[51] Rådström P, Swedberg G (1988). RSF1010 and a conjugative plasmid contain *sulII*, one of two known genes for plasmid-borne sulfonamide resistance dihydropteroate synthase. Antimicrob Agents Chemother.

[52] Hopkins KL, Davies RH, Threlfall EJ (2005). Mechanisms of quinolone resistance in *Escherichia coli* and *Salmonella*: recent developments. Int J Antimicrob Agents.

[53] Schwarz S, Kehrenberg C, Doublet B, Cloeckaert A (2004). Molecular basis of bacterial resistance to chloramphenicol and florfenicol. FEMS Microbiol Rev.

[54] Poirel L, Héritier C, Tolün V, Nordmann P (2004). Emergence of oxacillinase-mediated resistance to imipenem in *Klebsiella pneumoniae*. Antimicrob Agents Chemother.

[55] Cuzon G, Naas T, Bogaerts P, Glupczynski Y, Huang TD (2008). Plasmid-encoded carbapenem-hydrolyzing beta-lactamase OXA-48 in an imipenem-susceptible *Klebsiella pneumoniae* strain from Belgium. Antimicrob Agents Chemother.

[56] Nüesch-Inderbinen MT, Kayser FH, Hächler H (1997). Survey and molecular genetics of SHV beta-lactamases in Enterobacteriaceae in Switzerland: two novel enzymes, SHV-11 and SHV-12. Antimicrob Agents Chemother.

[57] Sutcliffe JG (1978). Nucleotide sequence of the ampicillin resistance gene of *Escherichia coli* plasmid pBR322. Proc Natl Acad Sci USA.

[58] Young H-K, Amyes SGB (1985). Characterisation of a new transposon-mediated trimethoprim-resistant dihydrofolate reductase. Biochem Pharmacol.

[59] Brisse S, Milatovic D, Fluit AC, Verhoef J, Martin N (1999). Comparative in vitro activities of ciprofloxacin, clinafloxacin, gatifloxacin, levofloxacin, moxifloxacin, and trovafloxacin against *Klebsiella pneumoniae*, *Klebsiella oxytoca*, *Enterobacter cloacae*, and *Enterobacter aerogenes* clinical isolates with alterations in GyrA and ParC proteins. Antimicrob Agents Chemother.

[60] Chopra I, Roberts M (2001). Tetracycline antibiotics: mode of action, applications, molecular biology, and epidemiology of bacterial resistance. Microbiol Mol Biol Rev.

[61] Ruzin A, Visalli MA, Keeney D, Bradford PA (2005). Influence of transcriptional activator RamA on expression of multidrug efflux pump AcrAB and tigecycline susceptibility in *Klebsiella pneumoniae*. Antimicrob Agents Chemother.

[62] Bratu S, Landman D, George A, Salvani J, Quale J (2009). Correlation of the expression of *acrB* and the regulatory genes *marA*, *soxS* and *ramA* with antimicrobial resistance in clinical isolates of *Klebsiella pneumoniae* endemic to New York City. J Antimicrob Chemother.

[63] Galimand M, Courvalin P, Lambert T (2003). Plasmid-mediated high-level resistance to aminoglycosides in Enterobacteriaceae due to 16S rRNA methylation. Antimicrob Agents Chemother.

[64] Chen YT, Lauderdale TL, Liao TL, Shiau YR, Shu HY (2007). Sequencing and comparative genomic analysis of pK29, a 269-kilobase conjugative plasmid encoding CMY-8 and CTX-M-3 beta-lactamases in *Klebsiella pneumoniae*. Antimicrob Agents Chemother.

[65] Martin P, Jullien E, Courvalin P (1988). Nucleotide sequence of *Acinetobacter baumannii aphA-6* gene: evolutionary and functional implications of sequence homologies with nucleotide-binding proteins, kinases and other aminoglycoside-modifying enzymes. Mol Microbiol.

[66] D'Andrea MM, Nucleo E, Luzzaro F, Giani T, Migliavacca R (2006). CMY-16, a novel acquired AmpC-type beta-lactamase of the CMY/LAT lineage in multifocal monophyletic isolates of *Proteus mirabilis* from northern Italy. Antimicrob Agents Chemother.

[67] Yong D, Toleman MA, Giske CG, Cho HS, Sundman K (2009). Characterization of a new metallo-beta-lactamase gene, *bla*_NDM-1_, and a novel erythromycin esterase gene carried on a unique genetic structure in *Klebsiella pneumoniae* sequence type 14 from India. Antimicrob Agents Chemother.

[68] Huovinen P, Huovinen S, Jacoby GA (1988). Sequence of PSE-2 beta-lactamase. Antimicrob Agents Chemother.

[69] Roberts MC (2005). Update on acquired tetracycline resistance genes. FEMS Microbiol Lett.

[70] Tribuddharat C, Fennewald M (1999). Integron-mediated rifampin resistance in *Pseudomonas aeruginosa*. Antimicrob Agents Chemother.

[71] Shen P, Wei Z, Jiang Y, Du X, Ji S (2009). Novel genetic environment of the carbapenem-hydrolyzing beta-lactamase KPC-2 among Enterobacteriaceae in China. Antimicrob Agents Chemother.

[72] Sundström L, Rådström P, Swedberg G, Sköld O (1988). Site-specific recombination promotes linkage between trimethoprim- and sulfonamide resistance genes. Sequence characterization of *dhfrV* and *sulI* and a recombination active locus of Tn21. Mol Gen Genet.

[73] Heikkilä E, Skurnik M, Sundström L, Huovinen P (1993). A novel dihydrofolate reductase cassette inserted in an integron borne on a Tn21-like element. Antimicrob Agents Chemother.

[74] Tennstedt T, Szczepanowski R, Braun S, Pühler A, Schlüter A (2003). Occurrence of integron-associated resistance gene cassettes located on antibiotic resistance plasmids isolated from a wastewater treatment plant. FEMS Microbiol Ecol.

[75] Briggs CE, Fratamico PM (1999). Molecular characterization of an antibiotic resistance gene cluster of *Salmonella typhimurium* DT104. Antimicrob Agents Chemother.

[76] Blickwede M, Schwarz S (2004). Molecular analysis of florfenicol-resistant *Escherichia coli* isolates from pigs. J Antimicrob Chemother.

[77] Weigel LM, Anderson GJ, Tenover FC (2002). DNA gyrase and topoisomerase IV mutations associated with fluoroquinolone resistance in *Proteus mirabilis*. Antimicrob Agents Chemother.

[78] Zienkiewicz M, Kern-Zdanowicz I, Gołebiewski M, Zyliñska J, Mieczkowski P (2007). Mosaic structure of p1658/97, a 125-kilobase plasmid harboring an active amplicon with the extended-spectrum beta-lactamase gene blaSHV-5. Antimicrob Agents Chemother.

[79] Hannecart-Pokorni E, Depuydt F, de Wit L, van Bossuyt E, Content J (1997). Characterization of the 6'-N-aminoglycoside acetyltransferase gene aac(6')-Im [corrected] associated with a sulI-type integron. Antimicrob Agents Chemother.

[80] Wang H, Guo P, Sun H, Wang H, Yang Q (2007). Molecular epidemiology of clinical isolates of carbapenem-resistant *Acinetobacter* spp. from Chinese hospitals. Antimicrob Agents Chemother.

[81] Vila J, Ruiz J, Goñi P, Jimenez de Anta T (1997). Quinolone-resistance mutations in the topoisomerase IV *parC* gene of *Acinetobacter baumannii*. J Antimicrob Chemother.

[82] Lee KY, Hopkins JD, Syvanen M (1990). Direct involvement of IS26 in an antibiotic resistance operon. J Bacteriol.

[83] Scoulica EV, Neonakis IK, Gikas AI, Tselentis YJ (2004). Spread of *bla_VIM-1_*-producing *E. coli* in a university hospital in Greece. Genetic analysis of the integron carrying the *bla_VIM-1_* metallo-beta-lactamase gene. Diagn Microbiol Infect Dis.

[84] Yigit H, Queenan AM, Rasheed JK, Biddle JW, Domenech-Sanchez A (2003). Carbapenem-resistant strain of *Klebsiella oxytoca* harboring carbapenem-hydrolyzing beta-lactamase KPC-2. Antimicrob Agents Chemother.

[85] Lauretti L, Riccio ML, Mazzariol A, Cornaglia G, Amicosante G (1999). Cloning and characterization of blaVIM, a new integron-borne metallo-β-lactamase gene from a *Pseudomonas aeruginosa* clinical isolate. Antimicrob Agents Chemother.

[86] Moura A, Soares M, Pereira C, Leitão N, Henriques I (2009). INTEGRALL: a database and search engine for integrons, integrases and gene cassettes. Bioinformatics.

[87] Papagiannitsis CC, Miriagou V, Kotsakis SD, Tzelepi E, Vatopoulos AC (2012). Characterization of a transmissible plasmid encoding VEB-1 and VIM-1 in *Proteus mirabilis*. Antimicrob Agents Chemother.

[88] Deguchi T, Fukuoka A, Yasuda M, Nakano M, Ozeki S (1997). Alterations in the GyrA subunit of DNA gyrase and the ParC subunit of topoisomerase IV in quinolone-resistant clinical isolates of *Klebsiella pneumoniae*. Antimicrob Agents Chemother.

[89] Sandegren L, Andersson DI (2009). Bacterial gene amplification: implications for the evolution of antibiotic resistance. Nat Rev Microbiol.

[90] Huang TW, Chen TL, Chen YT, Lauderdale TL, Liao TL (2013). Copy number change of the NDM-1 sequence in a multidrug-resistant *Klebsiella pneumoniae* clinical isolate. PLoS One.

[91] Walther-Rasmussen J, Høiby N (2006). OXA-type carbapenemases. J Antimicrob Chemother.

[92] Girlich D, Naas T, Nordmann P (2004). Biochemical characterization of the naturally occurring oxacillinase OXA-50 of *Pseudomonas aeruginosa*. Antimicrob Agents Chemother.

[93] Figueiredo S, Poirel L, Croize J, Recule C, Nordmann P (2009). In vivo selection of reduced susceptibility to carbapenems in *Acinetobacter baumannii* related to ISAba1-mediated overexpression of the natural *bla_OXA-66_* oxacillinase gene. Antimicrob Agents Chemother.

[94] Smillie C, Garcillán-Barcia MP, Francia MV, Rocha EP, de La Cruz F (2010). Mobility of plasmids. Microbiol Mol Biol Rev.

[95] Poirel L, Bonnin RA, Nordmann P (2012). Genetic features of the widespread plasmid coding for the carbapenemase OXA-48. Antimicrob Agents Chemother.

[96] Baker-Austin C, Wright MS, Stepanauskas R, McArthur JV (2006). Co-selection of antibiotic and metal resistance. Trends Microbiol.

[97] Poirel L, Dortet L, Bernabeu S, Nordmann P (2011). Genetic features of *bla_NDM-1_*-positive Enterobacteriaceae. Antimicrob Agents Chemother.

[98] Boulund F, Karlsson R, Gonzales-Siles L, Johnning A, Karami N (2017). Typing and characterization of bacteria using bottom-up tandem mass spectrometry proteomics. Mol Cell Proteomics.

[99] Andersson DI, Hughes D (2010). Antibiotic resistance and its cost: is it possible to reverse resistance?. Nat Rev Microbiol.

[100] EUCAST (2017). European Committee on Antimicrobial Susceptibility Testing. Data from The EUCAST MIC Distribution Website.

[101] Masuda N, Sakagawa E, Ohya S, Gotoh N, Tsujimoto H (2000). Substrate specificities of MexAB-OprM, MexCD-OprJ, and MexXY-oprM efflux pumps in *Pseudomonas aeruginosa*. Antimicrob Agents Chemother.

[102] Pai H, Kim J, Kim J, Lee JH, Choe KW (2001). Carbapenem resistance mechanisms in *Pseudomonas aeruginosa* clinical isolates. Antimicrob Agents Chemother.

[103] Walsh TR, Weeks J, Livermore DM, Toleman MA (2011). Dissemination of NDM-1 positive bacteria in the New Delhi environment and its implications for human health: an environmental point prevalence study. Lancet Infect Dis.

[104] Keeney D, Ruzin A, McAleese F, Murphy E, Bradford PA (2008). MarA-mediated overexpression of the AcrAB efflux pump results in decreased susceptibility to tigecycline in *Escherichia coli*. J Antimicrob Chemother.

[105] Livermore DM (2002). Multiple mechanisms of antimicrobial resistance in *Pseudomonas aeruginosa*: our worst nightmare?. Clin Infect Dis.

[106] Reuter S, Ellington MJ, Cartwright EJ, Köser CU, Török ME (2013). Rapid bacterial whole-genome sequencing to enhance diagnostic and public health microbiology. JAMA Intern Med.

[107] Dantas G, Sommer MO (2012). Context matters - the complex interplay between resistome genotypes and resistance phenotypes. Curr Opin Microbiol.

[108] Martínez JL, Coque TM, Baquero F (2015). What is a resistance gene? Ranking risk in resistomes. Nat Rev Microbiol.

[109] Liu YY, Wang Y, Walsh TR, Yi LX, Zhang R (2016). Emergence of plasmid-mediated colistin resistance mechanism MCR-1 in animals and human beings in China: a microbiological and molecular biological study. Lancet Infect Dis.

[110] Hu Y, Liu F, Lin IY, Gao GF, Zhu B (2016). Dissemination of the *mcr-1* colistin resistance gene. Lancet Infect Dis.

[111] Leclercq R, Cantón R, Brown DF, Giske CG, Heisig P (2011). EUCAST expert rules in antimicrobial susceptibility testing. Clin Microbiol Infect.

