# Supplementary material for: The resistomes of six carbapenem-resistant pathogens – a critical genotype–phenotype analysis
Source: Microb Genom. 2018 Nov 21;4(11):e000233. doi: 10.1099/mgen.0.000233 (PMC6321870; doi:10.1099/mgen.0.000233)
Supplement: Supplementary File 1 [file mgen-4-233-s001.pdf]

SUPPLEMENTAL MATERIAL

Table S1 Isolation data

Species	Strain	Isolation site	Treatment†	Treated abroad†
Pseudomonas aeruginosa	CCUG 70744	Sputum	Meropenem	-
Escherichia coli	CCUG 70745	Rectum	-	-
Klebsiella pneumoniae	CCUG 70742	Urine	-	-
Proteus mirabilis	CCUG 70746	Rectum	-	Balkan
Acinetobacter baumannii	CCUG 70743	Wound	-	Middle east
Klebsiella pneumoniae	CCUG 70747	Wound	Cefotaxime	Balkan

† No information marked with dash (-).

Table S2 Intrinsic resistance genes in the reference genomes excluded from further analysis

Strain	Gene	Function	Accession number
P. aeruginosa PAO1	*aph(3')-IIb*	Aminoglycoside phosphotransferase	X90856
	bla$_{OXA-50}$-like	β-lactamase	AY306130
	bla$_{PAO}$	β-lactamase	FJ666065
	catB7	Chloramphenicol acetyltransferase	AF036933
	fosA	Enzymatic fosfomycin resistance	NZ_ACWU01000146
K. pneumoniae MGH 78578	*bla*$_{SHV-11}$	β-lactamase	EF035558
	fosA	Enzymatic fosfomycin resistance	NZ_AFBO01000747
	oqxAB	Quinolone efflux pump	EU370913
P. mirabilis HI4320	*cat*	Chloramphenicol acetyltransferase	M11587
	tet(J)	Tetracycline efflux pump	ACLE01000065
A. baumannii ATCC 17978	*bla*$_{ADC-25}$	β-lactamase	EF016355
	bla$_{OXA-66}$	β-lactamase	FJ360530

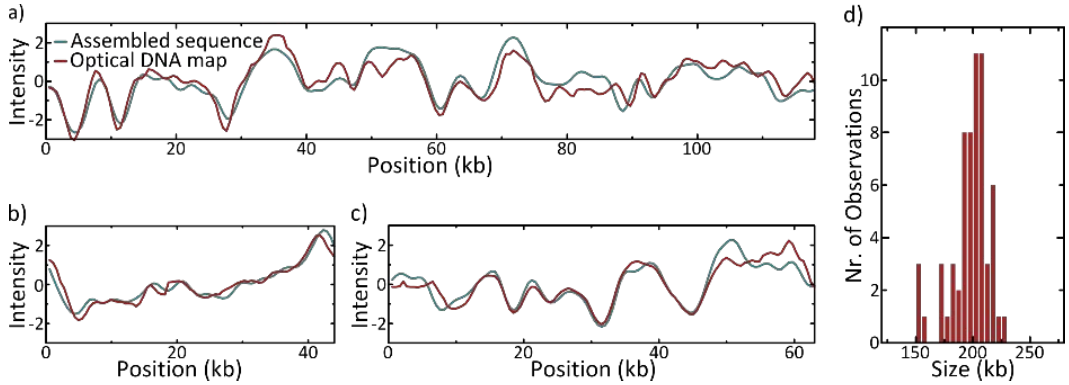

Fig. S1 Comparison between experimentally generated optical DNA maps (red) and theoretical optical DNA maps based on assembled sequences (teal) for non-novel plasmids **(a)** pKpn70747_2 in *K. pneumoniae* CCUG 70747 (118 kb), **(b)** pEco70745_2 in *E. coli* CCUG 70745 (44 kb), and **(c)** pKpn70742_2 in *K. pneumoniae* CCUG 70742 (63 kb). Intensity (y-axis) refers to the normalized emission intensity along the DNA molecule (x-axis) which correlates coarsely with the underlying DNA sequence. **(d)** Histogram displaying the size distribution of individual plasmid molecules found in *P. mirabilis* CCUG 70746, demonstrating a high variability of the plasmid within the sample.

Table S3 Chromosomal genes screened for mutations conferring resistance

Reference strain	Function	Gene	Locus tag
P. aeruginosa PAO1	Quinolone targets	*gyrA*	PA3168
		gyrB	PA0004
		parC	PA4964
		parE	PA4967
	β-lactamase	*ampC*	PA4110
	Penicillin-binding protein	*dacB*	PA3047
		dacC	PA3999
		ftsI	PA4418
		mrcB	PA4700
		pbpA	PA4003
		pbpC	PA2272
		pbpG	PA0869
		ponA	PA5045
	Drug efflux pumps	*mexA*	PA0425
		mexB	PA0426
		mexC	PA4599
		mexD	PA4598
		mexE	PA2493
		mexF	PA2494
		mexX	PA2019
		mexY	PA2018
		oprJ	PA4597
		oprM	PA0427
		oprN	PA2495
	Porin	*oprD*	PA0958
	Regulatory proteins	*ampD*	PA4522
		ampR	PA4109
		czcR	PA2523
		czcS	PA2524
		mexR	PA0424
		mexT	PA2492
		mexZ	PA2020
		nalC	PA3721
		nalD	PA3574
		nfxB	PA4600
E. coli str. K-12 substr. MG1655	Quinolone targets	*gyrA*	b2231
		gyrB	b3699
		parC	b3019
		parE	b3030
K. pneumoniae MGH 78578	Quinolone targets	*gyrA*	KPN_02640
		gyrB	KPN_03437
		parC	KPN_03444
		parE	KPN_04102
	Drug efflux pumps	*acrA*	KPN_00444
		acrB	KPN_00443
	Regulatory proteins	*acrR*	KPN_00445
		marA	KPN_01624
		marR	KPN_01625
		ramA	KPN_00556
		ramR	KPN_00555
		soxR	KPN_04463
		soxS	KPN_04462
	Outer membrane protein	*tolC*	KPN_03449
P. mirabilis HI4320	Quinolone targets	*gyrA*	HMPREF0693_1372
		gyrB	HMPREF0693_0114
		parC	HMPREF0693_2557
		parE	HMPREF0693_2558
A. baumannii ATCC 17978	Quinolone targets	*gyrA*	A1S_2626
		gyrB	A1S_0004
		parC	A1S_0194
		parE	A1S_3359

Table S4 MICs and EUCAST clinical resistance classifications

Antibiotic class	Antibiotic	*P. aeruginosa* CCUG 70744*		*E. coli* CCUG 70745*		*K. pneumoniae* CCUG 70742*		*P. mirabilis* CCUG 707466*		*A. baumannii* CCUG 70743*		*K. pneumoniae* CCUG 70747*	
Carbapenems	Ertapenem	>32	iR	>32	R	4	R	>32	R	>32	iR	>32	R
	Imipenem	>32	R	>32	R	0.5	S	>32	R	>32	R	>32	R
	Meropenem	>32	R	>32	R	0.5	S	32	R	>32	R	>32	R
Cephalosporins	Cefuroxime	>256	iR	>256	R	>256	R	>256	R	>256	iR	>256	R
	Cefotaxime	>32	iR	>32	R	>32	R	>32	R	>32	iR	>256	R
	Ceftazidime	256	R	>256	R	32	R	>256	R	>256	-	>256	R
	Ceftriaxone	>32	iR	>32	R	>32	R	>32	R	>32	iR	>32	R
Monobactams	Aztreonam	96	R	>256	R	64	R	2	I	>256	-	>256	R
Penicillins	Amoxicillin	>256	iR	>256	R	>256	R	>256	R	>256	iR	>256	R
	Ampicillin	>256	iR	>256	R	>256	iR	>256	R	>256	iR	>256	iR
	Amoxicillin/clavulanic acid	>256/2	iR	>256/2	R	>256/2	R	>256/2	R	>256/2	iR	>256/2	R
	Piperacillin/Tazobactam	>256/4	R	>256/4	R	256/4	R	32/4	R	>256/4	-	>256/4	R
Aminoglycosides	Amikacin	16	I	16	I	6	S	>256	R	64	R	32	R
	Gentamicin	4	S	1	S	2	S	>256	R	64	R	2	S
	Tobramycin	1	S	16	R	16	R	>256	R	64	R	16	R
Fluoroquinolones	Ciprofloxacin	1	I	>32	R	>32	R	>32	R	>32	R	>32	R
	Levofloxacin	8	R	>32	R	2	I	32	R	>32	R	32	R
	Moxifloxacin	8	R	>32	R	4	R	32	R	>32	-	>32	R
Sulfonamides	Trimethoprim/sulfamethoxazole	>32/608	iR	>32/608	R	>32/608	R	>32/608	R	>32/608	R	>32/608	R
Tetracyclines	Tetracycline	64	iR	>256	R	192	R	32	iR	>256	-	4	R
	Tigecycline	16	iR	1	S	4	R	32	R	8	-	1	S
Others	Colistin	0.5	S	0.25	S	2	S	>256	iR	1	S	2	S
	Fosfomycin	>256	-	0.25	S	32	S	8	S	1024	iR	64	R
	Chloramphenicol	>256	iR	64	R	4	S	256	R	>256	-	16	R

* S = susceptible, I = intermediate, R = resistant, iR = intrinsically resistant. Units are mg l^{-1}.

Table S5. Closest matching plasmids

Strain	Plasmid	Length (bp)	Closest match in GenBank	Length (bp)	Accession number	Isolation county	Alignment identity	Gaps	Same ARGs*
E. coli CCUG 70745	pEco70745_1	107 323	*Escherichia coli* isolate Co6114 plasmid pC06114_1	129 085	CP016035.1	Germany	67 %	19 %	No
	pEco70745_2	44 006	*Escherichia coli* strain OM26 plasmid pOM26-1	45 122	KP776609.1	Oman	98 %	3 %	Yes
	pEco70745_3	3 608	*Salmonella enterica* subsp. enterica serovar Typhimurium var. 5- str. CFSAN001921 plasmid unnamed	3 609	CP006052.1	USA	98 %	0 %	-
	pEco70745_4	1 934	*Escherichia coli* strain G3/10 plasmid pSYM3	1 934	KM107839.1	Germany	100 %	0 %	-
	pEco70745_5	1 780	*Escherichia coli* strain EC08 plasmid pEC08-1 RepA gene	1 980	JX238440.1	Sweden	93 %	0 %	-
	pEco70745_6	1 459	*Escherichia coli* strain ST648 plasmid pEC648_7	1 459	CP008721.1	China	100 %	0 %	-
K. pneumoniae CCUG 70742	pKpn70742_1	65 276	*Klebsiella pneumoniae* strain ATCC BAA-2146 plasmid pHg	85 161	CP006662.2	USA	67 %	26 %	No
	pKpn70742_2	63 075	*Enterobacter cloacae* strain INSRA17313-1 plasmid pUR17313-1	63 584	KP061858.1	Portugal	97 %	3 %	Yes
	pKpn70742_3	4 426	*Klebsiella pneumoniae* strain 0773 plasmid pKpn114, complete sequence	4 211	EU932690.1	China	96 %	7 %	-
	pKpn70742_4	1 780	*Escherichia coli strain* EC08 plasmid pEC08-1 RepA gene	1780	JX238440.1	Sweden	93 %	0 %	-
P. mirabilis CCUG 707466	pPmi70746_1	191 769	*Salmonella enterica* subsp. enterica serovar Corvallis plasmid pRH-1238	187 683	KR091911.1	Germany	78 %	20 %	No
A. baumannii CCUG 70743	pAba70743_1	10 880	*Acinetobacter baumannii* plasmid pAB120	10 879	JX069966.1	Lithuania	100 %	0 %	Yes
K. pneumoniae CCUG 70747	pKpn70747_1	186 310	*Klebsiella pneumoniae* JM45 plasmid p1	317 154	CP006657.1	China	52 %	41 %	No
	pKpn70747_2	117 859	*Klebsiella pneumoniae* strain Kpn-1870 plasmid pGR-1870	116 047	KF874498.1	Greece	89 %	7 %	Yes
	pKpn70747_3	32 773	*Salmonella enterica* subsp. enterica serovar Typhi linear plasmid pBSSB1	27 037	AM419040.1	Indonesia	73 %	21 %	-

* Antibiotic resistance genes (ARGs).

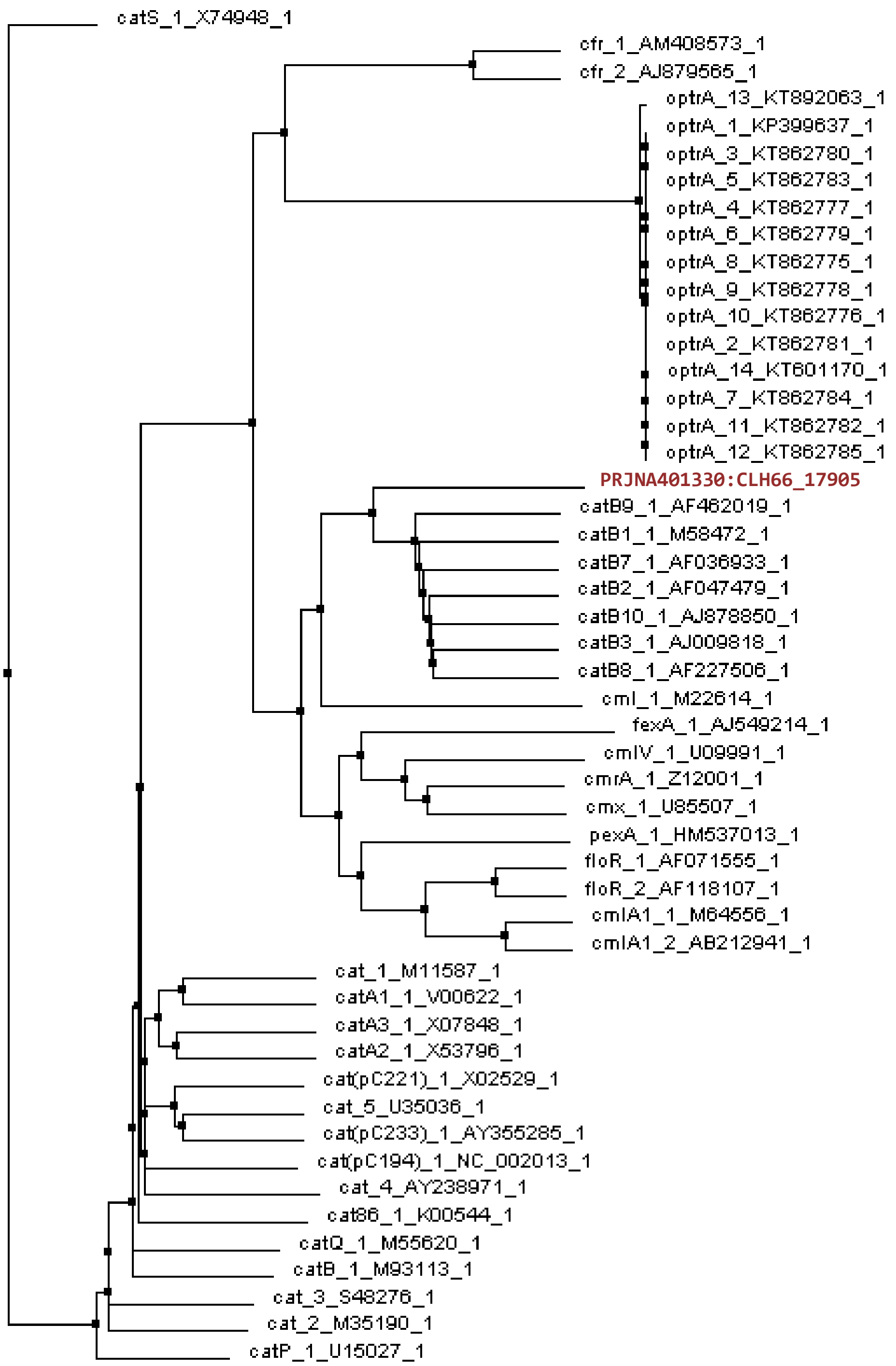

Fig. S2 (a) Multiple sequence alignment of all phenicol resistance proteins in the ResFinder database and the *cat*-like protein encoded by the chromosome of *E. coli* CCUG 70745. The putative phenicol resistance gene (protein id PRJNA401330:CLH66_17905) is most similar to the group of CatB enzymes. The alignment was done using Clustal Omega and the figure depicts a neighbour joining tree using the BLOSUM62 distance matrix.

```
> PRJNA401330:CLH66_17905
MKIMKINNIDVMKENDIVFSNTAGFWSGPVEIECPAEIRQGVYQVDKIGAF
SYMGGKSTHMLHIESIGRFCAIAGNVVAGAMEHPAHFLSPHPIMQGVFKWK
ALDDFKLKNKDMLWKSRTFNNQINKDRFGKIKIGNDVWIGEGAFIRRGVKI
GDGAIIASHSVVSKDVAPYSIVGGVPAKHIKFRFEDNIIEQLIEIKWWDYG
LSALEGVDFTDINKAIKKIRENIDSGVAEKFTPNVIRINKDGVAEKI*
```

Fig. S2 (b) The predicted protein sequence of the putative novel phenicol inactivating enzyme in *E. coli* CCUG 70745 .

```
AF462019_1      1 MNFFTSPFSGIPLDQQVTNPNIIVGKHSYYSGYYHGHSFDDCVRYLHPER     50
                  |...  ..:.|    .|...|.||  .|..:|::.|....:|...:.....
CLH66_17905     1 MKIM--KINNI----DVMKENDIV--FSNTAGFWSGPVEIECPAEIRQGV     42

AF462019_1     51 DDVDKL-----------------VIGSFCSIGSGAVFMMAGNQGHRSDWI     83
                  ..|||:                 .||.||:|....|   ||...|.:.::
CLH66_17905    43 YQVDKIGAFSYMGGKSTHMLHIESIGRFCAIAGNVV---AGAMEHPAHFL     89

AF462019_1     84 STFP-----FFYQDNDNFA-DARDGFTRS------------GDTIIGHDV    115
                  |..|     |.::..|:|. ..:|...:|            |...||:||
CLH66_17905    90 SPHPIMQGVFKWKALDDFKLKNKDMLWKSRTFNNQINKDRFGKIKIGNDV    139

AF462019_1    116 WIGTEAMIMPGVKIGHGAIIASRSVVTKDVAPYEVVGSNPAKHIKFRFSD    165
                  |||..|.|..|||||.||||||.|||:||||||.:||..|||||||||.|
CLH66_17905   140 WIGEGAFIRRGVKIGDGAIIASHSVVSKDVAPYSIVGGVPAKHIKFRFED    189

AF462019_1    166 VEIAMLLEMAWWNWPESWL------------KESMQSLCSSDIEG-----    198
                  ..|..|:|:.||::..|.|            |:..::.|...|.
CLH66_17905   190 NIIEQLIEIKWWDYGLSALEGVDFTDINKAIKKIRENIDSGVAEKFTPNV    239

AF462019_1    199 LYLNWQSKART-    209
                  :.:|....|..
CLH66_17905   240 IRINKDGVAEKI    251
```

Fig. S2 (c) A global protein alignment between the putative novel phenicol inactivating enzyme in *E. coli* CCUG 70745 (PRJNA401330:CLH66_17905) and the closest match in ResFinder, CatB9 (accession number AF62019) with 31.6% alignment identity, 42.6% similarity, and 24.7% gaps. Underlined region corresponds to the match against the conserved domain "Zenobiotic acyltransferase (XAT)" (see FIG. S2d).

```
                       10        20        30        40        50        60        70        80
                   ....*....|....*....|....*....|....*....|....*....|....*....|....*....|....*....|
CLH66_17905   48  IGAFSYMGGKSTHMLHI-ESIGRFCAIAGNVVAGA-MEHPAHFLSPHPIMQGVFKWKALDDFKlknkDMLWKsrtfnnqi 125
Cdd:cd03349   4   VGDYSYGSGPDCDVGGDkLSIGKFCSIAPGVKIGLgGNHPTDWVSTYPFYIFGGEWEDDAKFD----DWPSK-------- 71

                         90       100       110       120       130       140       150
                    ....*....|....*....|....*....|....*....|....*....|....*....|....*...
CLH66_17905   126 nkdrfGKIKIGNDVWIGEGAFIRRGVKIGDGAIIASHSVVSKDVAPYSIVGGVPAKHIKFRFEDNIIEQLIEIKWWDY 203
Cdd:cd03349   72  -----GDVIIGNDVWIGHGATILPGVTIGDGAVIAAGAVVTKDVPPYAIVGGNPAKVIRYRFDEETIERLLALKWWDW 144
```

FIG. S2 (d) Local alignment between the putative novel phenicol inactivating enzyme in *E. coli* CCUG 70745 (PRJNA401330:CLH66_17905) and the closest matching domain in the Conserved Domains Database (CDD): Zenobiotic acyltransferase (XAT) (Accession: cd03349; pssm-ID: 100040; domain length: 145; e-value: 6.30e-54). "The XAT class of hexapeptide acyltransferases is composed of a large number of microbial enzymes that catalyze the CoA-dependent acetylation of a variety of hydroxyl-bearing acceptors such as chloramphenicol and streptogramin, among others."
